# Extreme Tolerance of Nanoparticle‐Protein Corona to Ultra‐High Abundance Proteins Enhances the Depth of Serum Proteomics

**DOI:** 10.1002/advs.202413713

**Published:** 2025-01-22

**Authors:** Qiqi Liu, Mengjie Wang, Xin Dai, Shuangqin Li, Haoxiang Guo, Haozhe Huang, Yueli Xie, Chenlu Xu, Yuan Liu, Weihong Tan

**Affiliations:** ^1^ Zhejiang Cancer Hospital Hangzhou Institute of Medicine (HIM) Chinese Academy of Sciences Hangzhou Zhejiang 310022 China; ^2^ School of Molecular Medicine Hangzhou Institute for Advanced Study University of Chinese Academy of Sciences Hangzhou Zhejiang 310024 China; ^3^ Institute of Molecular Medicine (IMM) Renji Hospital Shanghai Jiao Tong University School of Medicine and College of Chemistry and Chemical Engineering Shanghai Jiao Tong University Hangzhou Shanghai 200240 China

**Keywords:** nanoparticle‐protein corona, nanoparticle‐protein interactions, protein binding affinity, protein spike‐in, triple‐protein assay

## Abstract

The serum nanoparticle‐protein corona (NPC) provides specific disease information, thus opening a new avenue for high‐throughput in‐depth proteomics to facilitate biomarker discovery. Yet, little is known about the interactions between NPs and proteins, and its role in enhanced depth of serum proteomics. Herein, a series of protein spike‐in experiments are conducted to systematically investigate protein depletion and enrichment during NPC formation. Proteomic depth is serum concentration‐dependent, and NPC exhibits powerful tolerance to ultra‐high abundant proteins. In addition, protein‐protein interactions (PPI), especially those involving albumin, play a pivotal role in promoting proteomic depth. Furthermore, a triple‐protein assay is established to interrogate the relationship between protein binding affinity and concentration. NPC formation is a product of balancing binding affinity, concentration, and PPI. Overall, this study elucidates how NPs achieve protein depletion and enrichment for enhanced serum proteomic depth to gain a better understanding of NPC as an essential tool of proteome profiling.

## Graphical abstract

The intrinsic tolerance of NPC to ultra‐high abundance plays an important role in serum NPC formation for enhanced proteomic depth, validated through spike‐in protein assay. The NPC formation is the result of a balance of protein binding affinity, concentration, and protein‐protein interactions.

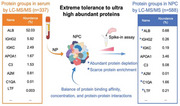

## 1 Introduction

The nanoparticle‐protein corona (NPC) is defined as a structure consisting of protein layers adsorbed onto nanoparticles (NPs) upon introduction into the biological milieu (e.g., blood, plasma, serum, bronchoalveolar lavage fluid, and urine).^[^ ^1^ ^]^ Formation of the protein corona is mainly driven by the physicochemical properties of NPs (e.g., nanoscale size, high surface curve, and surface energy).^[^ ^2^ ^]^ Typically, new biological identities of nanomaterials would be endowed through NPC formation, thereby shaping their biological fate, including biodistribution, cellular uptake, and therapeutic efficacy.^[^ ^3^ ^]^ Meanwhile, profiling biological identity through high‐throughput mass spectrometry opens a new avenue for biomarker discovery and disease diagnosis.^[^ ^4^ ^]^ Broad, deep, fast, and unbiased proteomics has already been achieved to interrogate complex biological samples, especially serum and plasma.^[^ ^5^ ^]^

Nano‐bio interactions are determined by electrostatic forces, van der Waals interactions, π–π stacking interaction, and hydrogen bonds.^[^ ^6^, ^7^ ^]^ Competition and replacement among proteins on the nano‐bio interface were observed and described by the Vroman effect,^[^ ^8^ ^]^ which characterizes the dynamic process of NPC formation and improves our understanding of the mechanisms underlying NPC formation.^[^ ^3^, ^9^ ^]^ It is well established that NPC formation is rapid and highly dynamic and that it is a product of the unique physiochemical properties of NPs, such as size, shape, porosity, surface modification and charge, and chirality,^[^ ^10^ ^]^ which significantly affect proteome profiles. Yet, little is known about the interactions between NPs and proteins, and its role in enhanced depth of serum proteomics.

Nanoproteomics takes advantage of NPC formation and high‐resolution mass spectrometry for rapid, deep, and unbiased proteomics. The wide range of serum protein concentrations spanning ≈10 orders of magnitude makes comprehensive profiling of serum extremely challenging.^[^ ^11^ ^]^ Conventionally, in‐depth proteomics can be achieved by protein fractionation and depletion of highly abundant protein species, but both are labor‐intensive and expensive, thus restricting proteomic studies to a small number of clinical samples. NPs can adsorb proteins owing to nanoscale size and intrinsic protein‐NP interactions.^[^ ^2^, ^12^, ^13^ ^]^ However, the use of NPCs, especially those derived from serum or plasma, represents a compelling tool to deplete high‐abundance proteins, but enrich low‐abundance proteins, for deep proteomics.^[^ ^14^ ^]^ NPC is being studied as a new way for biomarker discovery and cancer diagnosis.^[^ ^15^ ^]^ Using NPCs, we have developed a ProteoFish platform with in‐depth proteomics for the precise classification of benign and malignant small nodules of the lung.^[^ ^16^ ^]^ The Farokhzad team established a ProteoGraph platform that contains five distinct NPs for lung cancer biomarker discovery and diagnosis.^[^ ^4^, ^17^ ^]^ To date, protein corona‐based nanoproteomics has made substantial success in biomarker discovery, diagnosis, and prognosis of various diseases, such as lung cancer, ovarian cancer, Alzheimer's disease, pancreatic cancer, breast cancer, and classical papillary thyroid carcinomas.^[^ ^18^ ^]^ Some key publications have spent much effort to enhance the reproducibility and robustness of protein corona proteomics.^[^ ^19^ ^]^ Nevertheless, the fundamental factors that determine the profile of protein corona remain ambiguous. Therefore, understanding the fundamental driving force that NPC promotes proteomic depth, is critical to advancing the methodology and rational applications of this powerful breakthrough tool.

**Figure 1:**
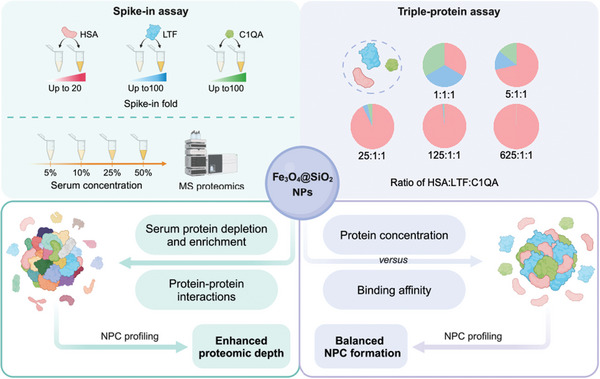
Schematic overview of deciphering enhanced proteomic depth of NPC via spike‐in assay and triple‐protein assay. Conducting spike‐in assay of three proteins of interest (HSA, LTF, and C1QA), together with 5%, 10%, 25%, and 50% serum concentrations to explain the intrinsic properties of NPC in protein depletion and enrichment and NP‐protein interactions (left). Establishing a triple‐protein assay to determine the balance of protein binding affinity and concentration in NPC formation (right). Created in https://BioRender.com.

To figure out the fundamental reason why NPC could enhance the proteomic depth, we herein first conducted a protein spike‐in assay to systematically investigate the intrinsic property of NPC in the protein depletion and enrichment and the tolerance to ultra‐high abundant proteins under different concentrations of serum (**Figure** **1**). PPI is the key to diverse corona proteins.^[^ ^20^ ^]^ We then established a triple‐protein assay to understand the balance struck among binding affinity, concentration, and PPI such that the product has a diverse proteomic profile. Through qualitative and quantitative analysis of protein corona profiles with liquid chromatography‐tandem mass spectrometry (LC‐MS/MS), we determined that 1) NPC shows the inherent property of depleting high‐abundance proteins and enriching low‐abundance proteins, 2) NPC possesses intrinsic tolerance to high‐abundance proteins in serum, and 3) protein binding affinity, concentration, and PPI, as well as the balance of all three determined the profile of NPC.

## 2 Results

### 2.1 Characterization of Serum Concentration‐Dependent NPC

**Figure 2:**
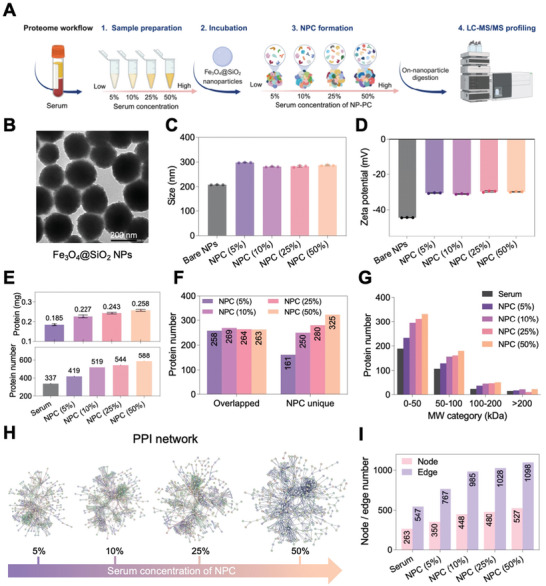
Serum concentration‐dependent Fe_3_O_4_@SiO_2_ NPC formation and proteomic profiling. A) Scheme of formation and profiling of NPC from different serum concentrations. B) TEM image of bare Fe_3_O_4_@SiO_2_ NPs. Scale bar, 200 nm. (C‐D) Hydrodynamic diameter (C) and zeta‐potential (D) of bare Fe_3_O_4_@SiO_2_ NPs and their NPCs in deionized water. Mean ± S.D., n = 3. E) Upper panel: protein amounts of Fe_3_O_4_@SiO_2_ NPCs (5%–50%). Mean ± S.D., n = 3. Values represent mean. Lower panel: the depth of proteomic profiling of pure serum and NPCs (5%–50%). F) Composition similarity analysis of proteins identified in serum and each NPC of different serum concentrations, respectively. G) Classification of corona proteins by molecular weight (MW). H) PPI network of corona proteins in NPCs (5%–50%) using STRING database. Minimum required interaction score = 0.900. Disconnected proteins are not shown. I) Node and edge number of each coronal PPI network in (H).

First, the impact of serum concentration on the formation of NPC was systemically investigated, as depicted in **Figure** **2**A. Fe_3_O_4_@SiO_2_ NPs were characterized by transmission electron microscopy (TEM) and dynamic light scattering (DLS). TEM images and measured hydrodynamic diameter revealed that Fe_3_O_4_@SiO_2_ NPs possessed a diameter of ≈200 nm (Figures 1B,C; Figures S1,S2, Supporting Information). After incubation with serum, the hydrodynamic diameter of Fe_3_O_4_@SiO_2_ NPCs increased from 208 to 298.3 nm (5%), 281.5 nm (10%), 283.0 nm (25%), and 287.4 nm (50%), which could be attributed to Brownian dynamics, which and describes the dynamics of molecular systems (NPs) in a diffusive regime (Figure 2C; Figure S2, Table S1, Supporting Information).^[^ ^21^ ^]^ Zeta‐potential measurements also indicated the elevated potentials of NPCs as serum concentration increases (Figure 2D).

The eluted proteins of Fe_3_O_4_@SiO_2_ NPCs were visualized using SDS‐PAGE gels (Figure S3A, Supporting Information), followed by semiquantitative densitometry analysis of gel bands (Figure S3B, Supporting Information). The amount and number of proteins in the NPCs are dependent on serum concentration, which is consistent with the gel band results (Figure 2E). The eluted protein amount is 0.185 mg (5%) which is far less than the total amount of protein in 5% serum (0.763 mg), indicating that the total protein in serum is far more than that adsorbed on the surface of NPs. The change in protein identification counts along serum concentration shows a trend similar to that of previous research based on the same regression model (Figure S4, Supporting Information).^[^ ^22^ ^]^ Notably, the number of NPC unique proteins showed a distinct increasing trend in a serum concentration‐dependent manner (Figure 2F; Figure S5, Supporting Information). In Figure 2G, protein characterization according to molecular weight (MW) revealed that NPC adsorbed more proteins with MW less than 50 kDa.

STRING is a biological database and web resource of known and predicted PPI.^[^ ^23^ ^]^ Here, we used the STRING database to further explore the biological network of corona proteins according to PPI (Figure 2H). We observed a broader and more condensed protein network because both edge and node numbers increased as serum concentration increased from 5% to 50% (Figure 2I). Such expansion of network complexity also correlates with the increased number of unique proteins, suggesting that the formation of NPCs was, at its core, facilitated by sophisticated biological PPI, in turn contributing to the increase of proteome coverage.

### 2.2 Enrichment of Low‐Abundance Proteins, but Depletion of High‐Abundance Proteins, to Boost Proteomic Depth

**Figure 3:**
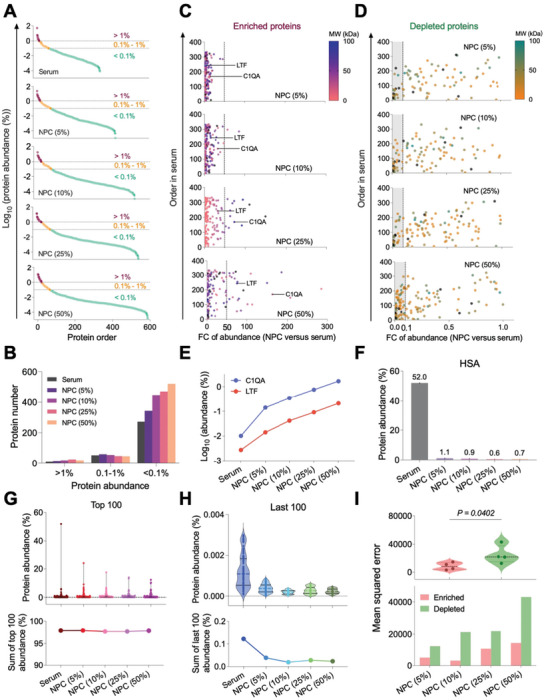
Low‐abundance protein enrichment and high‐abundance protein depletion of Fe_3_O_4_@SiO_2_ NPC for enhanced proteomic depth. A) Distribution of abundance of identified proteins in serum and NPCs of different serum concentrations by LC‐MS/MS. Abundance is represented as a percentage in Log10 and was divided into three levels. The red, orange, and light green populations represent the abundance of proteins as high (> 1%), middle (0.1%–1%), and low (< 0.1%), respectively. B) Quantity statistics of proteins in serum and NPCs categorized by abundance level. C,D) Overall abundance transition of enrichment and depletion. Fold change (FC) of abundance (NPC vs serum) of enriched proteins (C) and depleted proteins (D) in NPCs of different serum concentrations. Dashed lines represented a fifty‐fold increase in abundance for enriched proteins (C) or a 90% decrease in abundance for depleted proteins (D). The symbol color indicates the MW of each protein. Points in black represent proteins of MW larger than 100 kDa. E) Serum concentration‐dependent abundance transition of LTF and C1QA in serum and NPCs. F) Abundance of HSA in serum and NPCs. G,H) Top100 most (G) and least (H) abundant proteins in serum and NPCs at different serum concentrations. Upper: violin plot of protein abundance. Lower: sum of corresponding 100 proteins. I) Quantitative characterization of protein enrichment or depletion ability of NPC in different serum concentrations.

To further probe the protein enrichment and depletion underpinning the proteomic depth of NPC, we first analyzed the distribution of protein abundance (%) across three defined intervals (low: < 0.1%; medium: 0.1%–1%; high: >1%) (**Figure** **3**A). Low‐abundance proteins comprised the majority of proteins identified in both serum and NPCs (Figure 3B). Notably, the representation of low‐abundance proteins in NPCs showed a clear trend that increased with serum concentration. Specifically, the number of low‐abundance proteins in NPCs was 1.26 (5%), 1.63 (10%), 1.73 (25%), and 1.92 (50%) times higher than that of pure serum (Figure 3B), suggesting that higher proteomic coverage was mainly attributed to low‐abundance proteins. Moreover, the number of unique low‐abundance proteins in NPCs showed a significant increase from 175 (5%) to 351 (50%) (Figure S6, Supporting Information).

Subsequently, we classified the proteins into enriched (Figure 3C) and depleted groups (Figure 3D) based on their fold change (FC) values of NPC versus serum, thereby reflecting the relative protein abundance compared to serum. The highest FC value of enriched proteins demonstrated a clear trend of increase, starting from 46.3 for NPCs (5%) and escalating to 285.2 for NPCs (50%) (Figure 3C). As examples of enriched proteins, lactotransferrin (LTF) and complement C1q subcomponent subunit A (C1QA) continuously increased in proportion to serum concentration (Figure 3E). The lowest FC value for depleted proteins (Figure 3D) ranged from 0.0001 to 0.001 in the NPC group, resulting in a reduction in the abundance of serum proteins of over 99%. Human serum albumin, the most abundant protein accounted for 52.0% in serum but showed significant depletion in all NPCs (Figure 3F). The corresponding most abundant protein in NPCs accounted for 24.1% (5%), 17.6% (10%), 14.0% (25%), and 12.4% (50%), respectively, whereas the sum of the top 100 abundant proteins in serum and NPCs was almost equal (≈97.5%) (Figure 3G). In the case of the least 100 abundant proteins in serum and NPCs, the sum of protein abundance of each serumconcentration‐dependent NPC group was much less than that of the collective pure serum groups (Figure 3H). Furthermore, we quantified enriched and depleted proteins using mean squared error (MSE) (Figure S7, Supporting Information). All NPCs showed higher values in depleted proteins, indicating that protein depletion plays a more significant role than protein enrichment in contributing to the enhancement of serum proteome coverage (Figure 3I).

To test whether the protein enrichment and depletion could be extended to other materials, we compared SiO_2_, Fe_3_O_4_, and CeO_2_ three different nanomaterials but with the same size (200 nm). As shown in Figure S8 (Supporting Information), the corona proteome components vary along with different materials. The protein numbers are 581 for SiO_2_, 559 for Fe_3_O_4_, and 941 for CeO_2_ (Figure S8A, Supporting Information). They all showed high‐abundance protein depletion and low‐abundance protein enrichment phenomenon. The percentages of HSA are 2.6% for SiO_2_, 5.1% for Fe_3_O_4_, and 4.1% for CeO_2_ (Figure S8B, Supporting Information). Furthermore, similar distributions of serum proteins were observed in NPCs for all nanomaterials mentioned above and Fe_3_O_4_@SiO_2_ in which the MSEs of depleted proteins are higher than that of enriched proteins (Figure S9, Supporting Information). Therefore, the material has a significant effect on the corona component, and protein depletion and enrichment to boost proteomic depth could be applied to other nanomaterials, such as SiO_2_, Fe_3_O_4,_ and CeO_2_. Taking Fe_3_O_4_ as an example, we investigated the protein corona component under different concentrations of serum from 5% to 50%. The SDS‐PAGE gel image and its semiquantitative densitometry analysis showed obvious differences when different serum concentrations were used to form NPC with Fe_3_O_4_ (Figure S10, Supporting Information). To investigate the size and curvature effects on the NPC component, we compared the NPC profile of 200 nm SiO_2_ and 500 nm SiO_2_. As shown below in Figure S11A (Supporting Information), the total abundance of HSA is 2.5% for 200 nm SiO_2_ whereas the total abundance of HSA is 3.9% for 500 nm SiO_2_. In addition, the total number of proteins, the depleted proteins, and the enriched proteins are different between 200 nm SiO_2_ and 500 nm SiO_2_ (Figure S11B, Supporting Information). Furthermore, MW distribution in different NP sizes was also analyzed. Although the NP size has a significant effect on the protein corona component, no obvious difference in MW distribution was observed (Figures S11C,D, Supporting Information).

### 2.3 NPC Formation Resembles an Immune Reaction

While we established the profound effect of protein depletion versus enrichment on NPC formation, the fundamental driving force that accounts for such effect remained to be elucidated. To accomplish this, we looked more closely at proteins, which are the basic functional units for life, and even more closely at immune response, which is one of the most important functions of the host when challenged by the invasion of foreign organisms.^[^ ^24^ ^]^ Many serum proteins are involved in immune response, such as complement proteins and antibodies, and they work within the innate and adaptive immune systems to kill pathogens. For example, complement cascade can be activated by the binding of C1q to antibody‐antigen complexes.^[^ ^25^ ^]^ Therefore, upon introduction of NPs into serum, it is not surprising that adsorption of immune‐related serum proteins to NPC could be likened to a kind of in vitro mimicry of the immune response.

**Figure 4:**
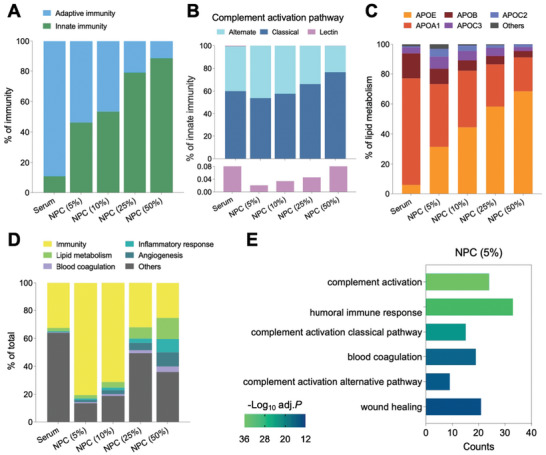
Profiling of immune‐related proteins of NPC. A) Composition analysis of adaptive and innate immunity in serum and NPCs (5%–50%). B) Composition analysis of innate immunity (complement activation pathway) in serum and NPCs (5%–50%). C) Composition analysis of lipid metabolism in serum and NPCs (5%–50%). D) Biological process‐based categorization of corona proteins, including immunity, lipid metabolism, inflammation, angiogenesis, and blood coagulation. E) Functional enrichment for NPCs (5%). The six most significant GO terms of the biological process were shown.

Notably, in humoral innate immune response, which consists of multiple components, including, for example, neutralizing antibodies and complement cascades, protein abundance increases with the serum concentration, whereas in the adaptive immune system, which also uses cells and proteins, such as immunoglobulins, to fight infection in response to called antigens, protein abundance decreases with the serum concentration (**Figure** **4**A). To validate this premise, we further analyzed three complement activation pathways of innate immunity, including classical, triggered by C1q binding to pathogen surface, alternative, triggered by antigen or C3 hydrolysis, respectively, and lectin through binding to the so‐called pathogen‐associated molecular patterns (PAMPs) on the surface of pathogens.^[^ ^26^ ^]^ As shown in Figure 4B, proteins associated with the classical pathway showed an increase in abundance, whereas proteins related to the alternative pathway displayed a decreasing trend along serum concentration. Proteins associated with the lectin pathway showed a slight increase. Immunoglobulins within the adaptive immune system, as noted above, showed a decreasing, but still relatively stable, pattern, with increasing serum concentration (Figure S12, Supporting Information). In addition, lipid metabolism, as a key regulator of T‐cell responses, is related to many immune‐related diseases.^[^ ^27^ ^]^ Strikingly, of the five major proteins in lipid metabolism, APOE, which mediates lipid transport and lipoprotein clearance, increased its NPC abundance with the increment of serum concentration, whereas APOA1, APOB, APOC2, and APOC3 all declined (Figure 4C). Collectively, these results suggest that various immune‐related proteins are involved in the NPC formation. Biological process (BP)‐based corona protein categorization indicated that immune‐associated proteins account for a major part of total proteins (Figure 4D). Furthermore, Gene Ontology (GO) enrichment analysis of NPCs showed the most enriched BPs for NPC (5%) were complement activation and humoral immune response (Figure 4E). Proteome profile analysis of SiO_2_, Fe_3_O_4_, and CeO_2_ NPs formed NPCs also indicated abundant immune‐related proteins including adaptive immunity and innate immunity (Figure S13, Supporting Information). In line with the biological immune response, these results tell us that introducing NPs to serum to form NPC resembles an immune reaction.

### 2.4 Intrinsic Tolerance of NPC to Ultra‐High Abundant Proteins

We previously demonstrated that NPC enhanced serum proteome coverage by depleting high‐abundance proteins and enriching unique, low‐abundance proteins. Here, we investigated the impact of high‐abundance proteins on NPC formation. To accomplish this, a triple‐protein series of spike‐in assays were carried out with three proteins of interest, including HSA, LTF, and C1QA, based on their baseline concentrations in pure serum. As noted previously, both C1QA and LTF were identified as low‐abundance proteins with potential binding advantages during NPC formation (Figure 2E), whereas HSA is a well‐established high‐abundance serum protein. Moreover, LTF and C1QA are both multifunctional proteins belonging to the innate immune system.^[^ ^28^ ^]^ Serum LTF is also a potential biomarker for allergic rhinitis, inherited thrombocytopenia 2, and severe aplastic anemia.^[^ ^29^ ^]^ C1QA plays a key role in activating complement cascade via the classic pathway as a primary activator, as suggested above. The abundance of C1QA in plasma will dramatically change the disease state and is involved in tumor microenvironment (TME) construction, immune modulation, and inflammation in cancer.^[^ ^30^ ^]^

In our spike‐in assay, HSA, as the most abundant protein in serum, was spiked in at levels up to 20‐fold. In contrast, LTF and C1QA, initially present at much lower concentrations (0.0028% and 0.0102%, respectively), were spiked in at levels up to 100‐fold to assess their relative quantities at different concentrations. SDS‐PAGE analysis of NPCs from these spike‐in assays revealed that HSA became more pronounced with increasing fold of spike‐in across all serum concentrations (Figure S14A, Supporting Information). However, enhanced band visibility of LTF and C1QA was primarily observed in NPCs derived from 50% serum concentration (Figures S14B,C, Supporting Information). Consequently, all spike‐in assays at 5% and 50% were further characterized through LC‐MS/MS.

**Figure 5:**
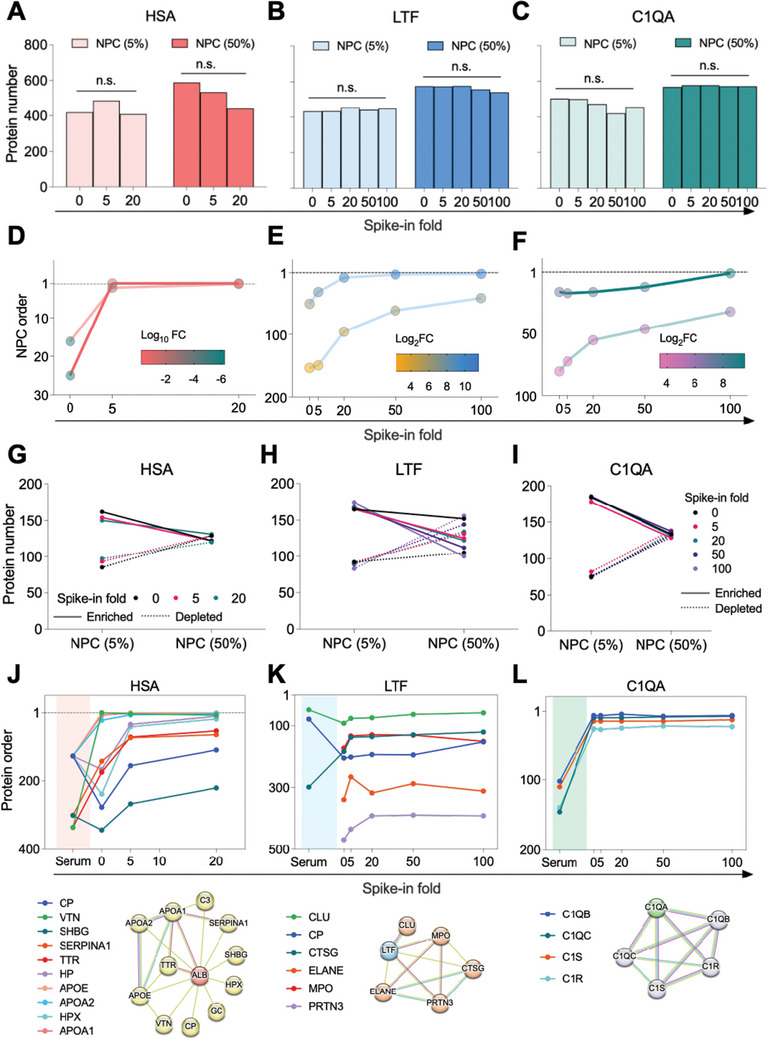
Spike‐in assay. A–C) Number of proteins identified in Fe_3_O_4_@SiO_2_ NPCs (5% and 50%) of HSA (A), LTF (B), and C1QA (C) spike‐in assay. P‐values are generated from the Wilcoxon sighed‐rank test. n.s. denotes non‐significance (P > 0.05). D–F) Order change of HSA (D), LTF (E), and C1QA (F) in NPCs (5% and 50%) before and after each protein spiking, respectively. Log2 FC of abundance (post‐spiking vs pre‐spiking) was indicated in the color bar. HSA was spiked in up to 20‐fold. LTF and C1QA were spiked up to the highest fold of 100‐fold. G–I) Serum concentration‐dependent regulation pattern in NPCs (5% and 50%) before and after HSA (G), LTF (H), and C1QA (I) spiking. J–L) Order change of proteins associated with HSA (J), LTF (K), and C1QA (L) in spiked‐in NPCs (50%) before and after corresponding protein spiking. Associated physical PPI subnetwork was shown below the corresponding line graph and was generated using the STRING database. Minimum required interaction score = 0.700.

Interestingly, although 5‐fold and even 20‐fold of HSA were spiked into serum at various concentrations, no significant change of proteome identification coverage by LC‐MS/MS was observed, indicating that NPC has an intrinsic tolerance to ultra‐high abundant proteins (**Figure** **5**A). The spike‐in of LTF and C1QA from 5‐fold to 100‐fold (5% and 50% of serum, respectively) did not change the protein identification coverage either, further demonstrating the tolerance and stability of NPC in serum protein coverage (Figures 5B,C). As expected, the abundance and rank of HSA, LTF, and C1QA in total proteins were increased as more spike‐in was added (Figures 5D–F; Figure S15, Supporting Information). Notably, even with spike‐in of LTF and C1QA, the phenomenon that high concentration of serum (50% vs 5%) results in better proteomic depth and NPC enrichment of more unique proteins are consistent with previous findings in this study (Figures 5A–C; Figure S16, Supporting Information). To test whether the tolerance to high‐abundance proteins could be extended to other nanomaterials, we also did HSA spike‐in to CeO_2_ NPs (Figure S17, Supporting Information). As more HSA was added to the serum, the abundance of HSA in protein corona increased from 10.4% (5‐fold) to 20.0% (20‐fold) (Figure S17B, Supporting Information). However, the number of protein types of NPC did not change obviously (Figure S17C, Supporting Information), confirming the intrinsic tolerance of CeO_2_ NPC to high‐abundance proteins.

However, these results did not directly address the behavior of NPC as a powerful tool to deplete high‐abundance proteins, but enrich low‐abundance proteins, for deep proteomics. Therefore, to investigate the influence of spike‐in proteins on protein depletion and enrichment, we further analyzed enriched and depleted proteins under different spike‐in conditions. As shown in Figure 5G, the number of enriched proteins is far more than that of depleted proteins. Spike‐in of HSA, whether 5‐fold or 20‐fold, did not change the number of enriched and depleted proteins much owing to the superior tolerance of NPC to spike‐in of HSA. However, the number of enriched proteins decreased as the serum concentration increased from 5% to 50%, whereas the number of depleted proteins increased in spike‐in of HSA, LTF, and C1QA, respectively (Figures 5G–I). Overall, our spike‐in experiment suggested that NPC exhibits a notable tolerance for high‐abundance proteins, including those proteins related to host immune response.

### 2.5 HSA Promotes the Corona Protein Diversity through Protein–Protein Interactions

HSA is the most abundant protein in serum. It is believed that HSA interferes with the identification of low‐abundance proteins in MS and the depletion of HSA will enhance the depth of serum proteomic. However, depletion of HSA is a double‐edge sword since it is also a very important carrier protein in serum.^[^ ^31^ ^]^ We believe that PPIs also play a pivotal role in enhancing serum proteomic depth during NPC formation. To test this notion, proteins associated with HSA, LTF, and C1QA were again analyzed, respectively (Figures 5J–L; Figure S18, Supporting Information). Both high‐abundance proteins, such as APOE and VTN, and low‐abundance proteins, such as CP, SHBG, HPX, and SERPINA1, showed an increment in protein rank as the spike‐in fold of HSA increased (Figure 5J; Figure S18A, Supporting Information). LTF and C1QA which are not carrier proteins do not have as many associated proteins as HSA. Only a few associated proteins such as PRTN3 and CP showed an increment trend along with spike‐in fold increase owing to PPI (Figures 5K,L; Figures S18B,C, Supporting Information). Our previous data indicated that HSA accounts for less than 5% in NPC, indicating that HSA was significantly reduced but not completely depleted (Figure 3F). To further elucidate the role of HSA in the case of CeO_2_ NPC, we profile the corona proteins of CeO_2_ NPC. As shown in Figure S19 (Supporting Information), some proteins, such as ANPEP, SHBG, and SPARC which may correlate with HSA, showed an increase as more HSA was introduced to serum. The PPI network confirmed that many proteins have significant correlations with HSA. Therefore, HSA could significantly promote the diversity of enriched protein corona as a carrier protein via PPI.

### 2.6 Binding Affinity, Protein Concentration, and Protein–Protein Interactions, as Determinants of Corona Protein Profiles

**Figure 6:**
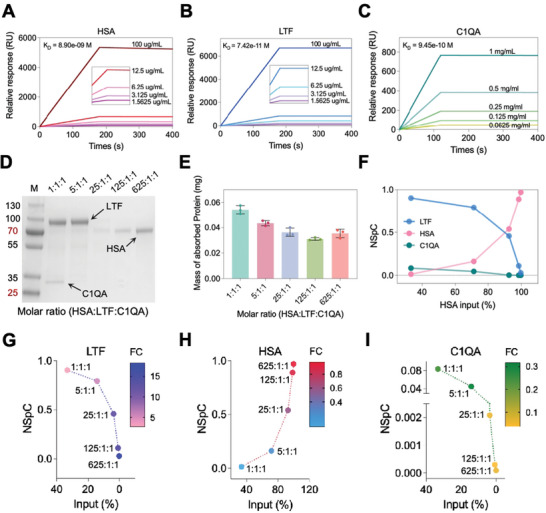
Protein competition reveals distinct binding modalities of proteins with different binding affinity. A–C) SPR response units (RU) as a function of time of NPs using immobilized HSA (A), LTF (B), and C1QA (C) as receptors. One site binding model was applied to fit the data and calculate the dissociation constants. Analysis indicated that Fe_3_O_4_@SiO_2_ NPs bind to HSA, LTF, and C1QA with dissociation constants KD = 8.9 nm for HSA, KD = 74.2 pM for LTF, and KD = 0.9 nM for C1QA. D,E) SDS‐PAGE gel (D) and mass (E) of proteins retrieved from NPCs of the triple‐protein assay. F) Normalized spectral count (NSpC) of HSA (pink), LTF (blue), and C1QA (green) as a function of HSA input (%). G–I) Multiple variables analysis of LTF (G), HSA (H), and C1QA (I). The color of bubbles gradated based on FC value (NSpC vs input).

To gain further insight into the binding feature of the mentioned proteins, the binding affinity of each protein to NPs was detected using SPR. The order of binding affinity from high to low was LTF (KD = 74.2 pm), C1QA (KD = 0.9 nM), and HSA (KD = 8.9 nM) (**Figures** **6**A–C). In addition, we established a triple‐protein assay to interrogate the relationship of protein binding affinity and concentration during the formation of NPC. For the mono‐protein assay, the quantities of adsorbed proteins of NPCs were 0.63 nmol (LTF), 0.52 nmol (C1QA), and 0.48 nmol (HSA) (Figure S20, Supporting Information). The order of adsorbed protein quantities is well matched with the binding affinity of the corresponding proteins, indicating that binding affinity determines protein adsorption to NPs.

Then, considering that HSA is the most abundant protein in serum,^[^ ^32^ ^]^ we set the total protein input as 1 nmol with LTF equal to C1QA in the triple‐protein model. Starting from HSA:LTF:C1QA = 1:1:1, HSA was increased from 33.3% to 71.4% (5:1:1), 92.6% (25:1:1), 98.4% (125:1:1), and 99.7% (625:1:1), respectively. Thus, from the ratio 1:1:1 to 5:1:1, the binding affinity appears to play a major role in the formation of NPC. Obvious bands of LTF and C1QA were observed in SDS‐PAGE (Figure 6D). However, when HSA was increased from 92.6% to 98.4% and 99.7%, concentration was observed to play a major role in the formation of NPC since the HSA band became more prominent. Figure 6E indicated that the mass of adsorbed proteins decreased gradually and then reached a plateau. This could be attributed to the high binding affinity and high MW of LTF at the beginning. When HSA dominates the input, the mass of adsorbed proteins will no longer decrease. Therefore, our triple‐protein model demonstrated the correlation of binding affinity and concentration in the formation of NPC.

To define the composition of NPCs, the amount of protein adsorbed onto (bound to) NPC was characterized with LC‐MS/MS and quantified as normalized spectral count (NSpC), a percentage calculated by normalizing spectral counts of identified proteins by using the respective MW.^[^ ^33^ ^]^ As shown in Figure 6F, LTF accounts for 90.34% of the NSpC at the ratio of 1:1:1 because of its high binding affinity and MW. Although C1QA has a lower MW than that of HSA, the NSpC of C1QA was still higher than that of HSA owing to its higher binding affinity. However, as the input of HSA increased from 33.3% to 99.7%, the adsorption of HSA in NPC significantly increased, whereas the adsorption of LTF and C1QA decreased. This result indicated that concentration would dominate the profile of NPC when HSA is extremely high regardless of the binding affinity.

To comprehensively compare the dynamic behavior of competitive adsorption of the three proteins, FC value (NSpC vs input) was calculated. Notably, the FC of LTF is always > 1, regardless of its input, indicating that LTF is an enriched protein (Figure 6G). However, the FC of HSA is always < 1, indicating that HSA is a depleted protein (Figure 6H). Similarly, the FC of C1QA in Figure 6I indicates that it is a depleted protein, even though it was an enriched protein in serum (Figure 3C). In summary, protein enrichment or depletion is the result of balancing binding affinities, concentration, and PPI. Proteins with high binding affinity are typically enriched proteins at low concentrations, whereas proteins with low binding affinity are depleted proteins at high concentrations.

## 3 Discussion

Previous studies, including those citing the Vroman effect that describes the process of competitive protein adsorption to a surface by blood serum proteins, have thoroughly reported on NPC formation.^[^ ^1^, ^9^ ^]^ Therefore, we know that materials, size, and surface modification of NPs affect the diversity of corona proteins.^[^ ^6^, ^34^ ^]^ Protein adsorption is a dynamic process in which an equilibrium is reached after 1 h when NPs are introduced into serum or plasma.^[^ ^9c^ ^]^ Usually, proteins with higher concentrations in serum would be adsorbed onto the surface first and gradually be replaced by proteins with higher binding affinity. A higher concentration of serum often results in more proteins adsorbed onto the surface of NPs. Due to complex ligand‐protein interactions, different ligands on the NP surface typically have distinct preferences for protein adsorption.^[^ ^35^ ^]^ Thus, ligand modification is often applied to achieve targeting interactions. It has also been reported that tuning the ratio of protein to NPs could identify more proteins.^[^ ^22^ ^]^ However, considering the concentration and complexity of serum, the fundamental factors that determine the profile of protein corona remain ambiguous. Previous studies have done a thorough exploration in the effects of concentration, binding affinity, and PPI independently.^[^ ^36^ ^]^ Here, we systematically investigated the role of serum concentration, protein binding affinity, as well as the PPI, in the formation of NPC to elucidate how balance among these factors results in enhancing NPC serum proteome coverage by depleting high‐abundance proteins and enriching low‐abundance proteins.

The formation of NPC resembles an immune reaction. Various proteins linked to both innate and adaptive immunity which consists of neutralizing antibodies, complement cascades, immunoglobulins, etc., were adsorbed on the surface of NPs being introduced into serum. Notably, these proteins showed a concentration‐dependent manner. Thus, abundant immune‐related serum proteins in protein corona could be likened to a kind of in vitro mimicry of the immune response. Our results demonstrated that NPC enhanced serum proteome coverage by depleting high‐abundance proteins and enriching low‐abundance proteins. For example, HSA alone accounts for ≈50% of total serum proteins, but a spike‐in of excess HSA to serum to 5‐fold, or even 20‐fold, did not substantially change serum proteome coverage with NPC by the depletion of this abundant protein. NPC could reduce the overwhelmed HSA in complicated serum owing to its intrinsic tolerance to ultra‐high abundant proteins. Furthermore, Spike‐in of LTF and C1QA also showed consistent depletion of high‐abundance proteins and enrichment of low‐abundance proteins. Therefore, together with HSA‐protein interactions and other PPI, a deep proteome coverage of NPC could be maintained regardless of high‐abundance proteins.

To investigate the influence of binding affinity and concentration of proteins in NPC formation, we established a mono‐protein assay and triple‐protein assay. In mono‐protein assay, the quantity of protein adsorption was solely determined by protein binding affinity. In triple‐protein assay, binding affinity dominates the profile of NPC when there is no high‐abundance protein. As the high‐abundance protein gets prominent, concentration will replace binding affinity and dominate the protein adsorption. In the case of serum which is more complex, protein binding affinity, concentration, and PPI will compete with each other in different proteins. Stable NPC with diversified proteins will be obtained when the equilibrium of the competition reaches. Thus, the formation of NPC is determined by protein binding affinity, concentration, and PPI, as well as the balance of all three.

Besides proteomic depth, the physiological character study of protein corona is very critical in nanomedicine for personalized precision medicine.^[^ ^37^ ^]^ Plasma from different individuals have variances in protein types and concentrations which will significantly influence the protein corona characters.^[^ ^38^ ^]^ Our protein spike‐in study also advances the understanding of personalized protein corona. Future exploration in this dimension will provide valuable insights into the variability of protein corona compositions among different individuals, thereby broadening the applicability and impact of the clinical transformation of nanomedicine.^[^ ^39^ ^]^

Finally, this study has some limitations. First, proteins are not the only bio‐molecules that adsorb NPs.^[^ ^40^ ^]^ It has been reported that cholesterol and phosphatidylcholine could modulate the formation of NPC.^[^ ^7^, ^41^ ^]^ Other biomolecules, such as metabolites, lipids, DNA, and RNA, may also influence the composition of NPC.^[^ ^42^ ^]^ Therefore, future studies may address how nucleic acid, metabolites, and lipids mediate the formation of NPC. Second, we believe that the formation of NPC is biofluid‐dependent. Cerebrospinal fluid, urine, saliva, and tears are different from serum in protein concentration, abundance, and complexity. Therefore, exploring the proteome coverage of NPC with other biofluids could serve as a potential source of new biomarkers and aid in more accurate disease diagnosis.^[^ ^11^, ^17^, ^43^ ^]^

## 4 Conclusion

In conclusion, we deciphered that 1) higher serum proteomic coverage of NPC is attained through the depletion of high‐abundance proteins and the enrichment of low‐abundance proteins, 2) NPC possesses intrinsic tolerance to high‐abundance proteins in complex serum, and 3) protein binding affinity, concentration, and PPI, as well as the balance of all three determined the formation of NPC in serum. Through our characterization of fundamental NPC formation, we provide insights that widen the application of in‐depth NPC proteomics for biomarker discovery and disease diagnosis.

## 5 Experimental Section

### Reagents

Iron (III) chloride hexahydrate (FeCl_3_·6H_2_O), trizma hydrochloride, DL‐dithiothreitol (DTT), iodoacetamide (IAA), and acetonitrile (gradient grade, ≥ 99.9%) were purchased from Sigma–Aldrich (Steinheim, Germany). Sodium acetate anhydrous (NaAc) was obtained from Macklin, Inc. (Shanghai, China). Tetraethyl orthosilicate (TEOS), ethanol, ammonium hydroxide (> 28% NH_3_ in H_2_O), and sodium citrate were purchased from Aladdin Biochemical Technology Co., Ltd. (Shanghai, China). Pierce BCA Protein Assay Kit (catalog number 23225), Trifluoroacetic acid (TFA), Formic acid (FA), and Pierce C18 Tips (100 µL) were purchased from Thermo Fisher Scientific. HSA and Coomassie Brilliant Blue for SDS‐PAGE were purchased from Solarbio (Beijing, China). LTF and C1QA were purchased from Uscn Life Science Inc. (Wuhan, China). Ultrapure Milli‐Q water with a resistivity of 18.2 MΩ was used for the preparation of solutions. All serum samples were approved by the Ethics Board of Zhejiang Cancer Hospital (IRB‐2022‐120).

### Synthesis and Characterization of Fe_3_O_4_@SiO_2_ NPs

Fe_3_O_4_@SiO_2_ NPs were synthesized according to published methods with some modifications.^[^ ^44^ ^]^ In brief, Fe_3_O_4_ NPs were synthesized according to the reported literature via solvothermal reaction. To produce spherical Fe_3_O_4_ NPs, 0.01 mol of FeCl_3_·6H_2_O was first dispersed into absolute ethanol (60.0 mL). Then, sodium citrate (1.00 g) and NaAc (4.20 g) were added and stirred vigorously for several minutes. Subsequently, the mixture was transferred into a sealed Teflon‐lined stainless‐steel autoclave with a capacity of 100.0 mL and heated at 200 °C for 10 h. The color of this mixture turned yellow‐brown and finally became quite black. After cooling to room temperature, the mixture was washed with ethyl alcohol and ultrapure water 3 times to eliminate organic and inorganic byproducts. The collected sediment was dispersed into deionized water and stored at 4 °C.

Na_2_SiO_3_ was used to fabricate the core‐shell Fe_3_O_4_@SiO_2_ structure. With continuous stirring, 0.5 g of prepared Fe_3_O_4_@SiO_2_ NPs were first dispersed in distilled water (10 mL). The resulting mixture was transferred into absolute ethanol (400 mL) and subjected to ultrasonication for 30 min. Subsequently, ammonia (10.0 mL) was rapidly introduced to the solution and stirred for 10 min at 610 rpm. Thereafter, TEOS (3.0 mL) was added, and the reaction was conducted for 3.5 h in the absence of light. Lastly, the products were collected using a strong magnet and further washed with ultrapure water and anhydrous ethanol (3 times for each), resulting in the formation of Fe_3_O_4_@SiO_2_ NPs.

### Characterization of NPs

The morphology of Fe_3_O_4_ NPs and Fe_3_O_4_@SiO_2_ NPs was performed with the JEM‐F200 Field Emission Transmission Electron Microscope (TEM) (JEOL, Japan) and the use of copper‐coated carbon TEM grids with an accelerating voltage of 200 kV. The shell thickness of NPs was measured by ImageJ software. Measurement of hydrodynamic diameter and zeta potential of synthesized Fe_3_O_4_@SiO_2_ NPs was characterized by DLS (Zetasizer Nano ZS ZEN3600, Malvern, United Kingdom). The solution of Fe_3_O_4_@SiO_2_ NPs was diluted to a concentration of 200 µg mL^−1^ (using 18 mΩ water) and sonicated 5 min before testing. Both tests were measured at 25 °C after 30 s equilibration. All reported DLS, PDI, and zeta potential values were averaged from 3 individual tests.

### NPC Formation and Characterization

2 mg Fe_3_O_4_@SiO_2_ NPs (100 µL) were incubated with serum at the following serum concentrations: 5%, 10%, 25%, and 50% for 1 h incubation with continuous shaking at 25 °C. After incubation, washing with washing buffer (150 mM NaCl) was performed twice. A magnet was used to separate extra serum or liquids from NPCs or NPs. Then, 2% SDS solution was added to suspend the NPC complex, and coronal proteins were collected in SDS solution after heating and ultrasound for future SDS polyacrylamide gel electrophoresis (SDS‐PAGE), BCA quantification, or use in deionized H_2_O for size distribution and zeta potential characterization by DLS. For SDS‐PAGE, supernatants of eluted protein solution were mixed with sample loading buffer followed by 10 min of heating. Gels were stained using 0.025% (w/w) Coomassie Brilliant Blue for 30 min and washed in deionized water until the background was clean.

### Spike‐In Assay

The baseline concentration of HSA, LTF, and C1QA in the serum sample was calculated by multiplying protein abundance (%) by serum concentration. Stock solution and appropriate dilutions of each protein were prepared and spiked into the serum samples of different concentrations. The final concentration of HSA reached 5‐fold and 20‐fold higher than baseline endogenous concentrations, while the final concentration of both LTF and C1QA were increased by 5‐, 20‐, 50‐, and 100‐fold. The volume of additions to the pooled serum was lower than 15% of the total sample volume. The samples were further incubated with Fe_3_O_4_@SiO_2_ NPs to form NPC and proteomic profiling to evaluate the tolerance of Fe_3_O_4_@SiO_2_ NPs to high‐abundance proteins as described in the Results.

### Triple‐Protein Assay

The incubation system included 100 µL solution containing total proteins of 1 nmol and 100 µL Fe_3_O_4_@SiO_2_ NPs of 0.5 mg for best recovery ratio and rational cost. For more accurate quantification results, the amount of protein bound to NPs was measured using Qubit fluorometers and Qubit Protein BR Assay Kit (Thermo Fisher Scientific), following the manufacturer's protocol. For the mono‐protein assay, 1 nmol HSA, LTF, and C1QA were incubated with Fe_3_O_4_@SiO_2_ NPs, respectively. For the triple‐protein assay, the input ratio of HSA:LTF:C1QA in molar was set as: 1:1:1, 5:1:1, 25:1:1, 125:1:1, and 625:1:1. In other words, HSA accounts for 33.3%, 71.4%, 92.6%, 98.4%, and 99.7% of input protein, while at the same time, the percentage of LTF and C1QA decreased from 33.3% to 14.3%, 3.7%, 0.79%, and 0.16%, respectively. The protein mixture was incubated with Fe_3_O_4_@SiO_2_ NPs to form NPC using the same procedure as mentioned above.

### Sample Preparation and Proteomic Profiling

All procedures were previously published by our group. On‐nanoparticle digestion was performed to digest proteins bound onto Fe_3_O_4_@SiO_2_ NPs. In brief, NPC complexes were dissolved in 30 µL of lysis buffer containing 10 mm dithiothreitol (DTT) and heated at 95 °C for 10 min. After cooling to room temperature, the proteins were then alkylated with 20 mM iodoacetamide (IAA) for 30 min in the dark. Subsequently, digestion was performed with trypsin at 37 °C with continuous shaking overnight and quenched the next day with 1% trifluoroacetic acid (TFA). A total of 30 µg of digestion mixture was loaded on Pierce C18 Pipette Tips (Thermo Fisher Scientific) according to the manufacturer's instructions. The sample was stored at −80 °C before LC‐MS/MS analysis.

### Data‐Independent Acquisition (DIA) Analysis

For DIA analysis, 2 µL (500 ng) of eluted peptides were reconstituted in a solution of 0.1% FA. First, the peptides were loaded into a trap column and then separated on an analytical column (75 µm i.d. × 25 cm, Thermo Fisher Scientific) using a standard preset gradient method. The 80 min gradient was set as follows: 3%–45% buffer B in 62 min, 45%–95% buffer B for 2 min, followed by a 16 min 95% wash. The parameters for full‐scan MS were as follows: the resolution of 60000 across 400–2000 m/z; DIA scans with a resolution of 30000; AGC target: 1e^6^, and maximal injection time of 22 ms. For MS acquisition, the isolation window (center of isolation window) was set to 30 variables in (m/z) as follows: 363.5, 388.5, 405, 419, 432, 444.5, 458, 472, 485, 497.5, 510.5, 524, 537, 550.5, 566.5, 584.5, 602.5, 618.5, 634.5, 651.5, 670.5, 691, 713.5, 742.5, 776.5, 808.5, 841, 890, 965.5, and 1156. The DIA raw files were processed on Spectronaut with default settings. The MS/MS spectra were searched against the spectral library generated by our group.^[^ ^16a^ ^]^ Data filtering was set to “Qvalue”, and FDR was set to 1% at both the protein and precursor levels. Peptide and protein reports were exported as CSV files for subsequent statistical analysis and visualization.

### Protein Data Preprocessing

Differential analysis was performed using R software. First, the significantly enriched proteins in Fe_3_O_4_@SiO_2_ NPs NPCs are selected. Screening criteria were as follows: fold change (FC) ≥ 1.2 or ≤ 0.83 and FDR‐adjusted P‐value of < 0.05. P‐values were adjusted using the Benjamini–Hochberg method. Functional enrichment analyses were performed according to Gene Oncology (https://www.geneontology.org). The org.Hs.eg.db and clusterProfiler packages in R were used to carry out GO analyses. Among the three GO terms (i.e., biological processes (BP), molecular function, and cellular component), BP was the most variable part and the six most significantly enriched BPs were selected to exhibit according to adjusted P‐value.

To quantify the ability of Fe_3_O_4_@SiO_2_ NPs in serum protein depletion and enrichment, mean squared error (MSE) was used and calculated by Wolfram Mathematica 12.1.0.0 software. Y = X was the predicted regression line, and thus the equation with a little modification was as follows:

(1) $$\mathrm{MS}E_{i}=\frac{1}{n}\;\sum_{i=1}^{n}{\left(Y_{i}-X_{i}\right)}^{2}$$

For triple‐protein assay, NSpC was used to describe the composition of NPC for better accuracy and was calculated as follows:

(2) $$\mathrm{NSp}C_{i}=\frac{\mathrm{Sp}C_{i}/MW_{i}}{\sum_{i=1}^{n}{\left(\mathrm{SpC}/\mathrm{MW}\right)}_{i}}\;$$

### SPR

BiacoreTM 8K SPR equipment was used to quantitatively characterize binding interactions between Fe_3_O_4_@SiO_2_ NPs and three proteins. Biacore Insight software was applied for complete instrument control and data evaluation. HSA and LTF were measured on the CM5 sensor chip, while C1QA was measured on the NTA chip. In brief, all proteins as ligands were dissolved in 10 mm sodium acetate buffer (pH 4.0). Then, NPs as analytes were diluted in PBS to the desired concentration. The dissociation constants (KD) were calculated using a 1:1 binding model by the software mentioned above.

### Statistical Analysis

GraphPad Prism 9 (GraphPad Software, USA) was utilized for statistical analysis and data visualization. MSE values were calculated by using Wolfram Mathematica 12.1.0.0 (Wolfram Research, Inc., Champaign, IL, USA) (Figure 3I; Figure S9, Supporting Information). The abundance (%) of identified proteins was expressed as a percentage and proteins with abundance (%) below 0.1% were considered as low‐abundance proteins. The protein abundance was transformed into Log 10 form for better visualization in Figure 3A and transformed into Log2 form in Figures 3E and 5D–F. Normalization of gray values of NPC was made by defining the smallest and the largest values in each NPC dataset as 0 and 1, respectively (Figures S3B,S10B, Supporting Information). The NSpC (Figures 6F–I) was normalized by setting the sum of group values as 1. Characterization of NPs and NPCs (Figures 2C–E; Figures S1‐S3,S10, Table S1, Supporting Information), mono‐protein assay (Figure S20, Supporting Information), and triple‐protein assay (Figure 6E), as well as the measurement of serum concentration (Figure S4A, Supporting Information) were conducted in replicates with sample size (n) indicated, and data were shown as mean ± S.D. The statistical test P‐values for significant difference results were directly indicated in the corresponding figure legend, denoted as n.s. (non‐significant). Differences between groups were considered statistically significant when P‐value < 0.05. Comparison between groups was made using paired t‐test analysis (Figure 3I). The comparison of proteomic depth in spike‐in assay was obtained using the Wilcoxon signed‐rank test (Figures 5A–C; Figure S17C, Supporting Information).

## Conflict of Interest

The authors declare no conflict of interest.

## Supporting information



Supporting Information

Supplemental Table 1

## Data Availability

The data that support the findings of this study are available in the supplementary material of this article.
