# Supplementary material for: Extreme Tolerance of Nanoparticle‐Protein Corona to Ultra‐High Abundance Proteins Enhances the Depth of Serum Proteomics
Source: Adv Sci (Weinh). 2025 Jan 22;12(11):2413713. doi: 10.1002/advs.202413713 (PMC11923864; doi:10.1002/advs.202413713)
Supplement: Supplementary file 1 — Supporting Information [file ADVS-12-2413713-s001.docx]

Supporting Information

Extreme Tolerance of Nanoparticle-Protein Corona to Ultra-High Abundance Proteins Enhances the Depth of Serum Proteomics

Qiqi Liu,^†^ Mengjie Wang,^†^ Xin Dai, Shuangqin Li, Haoxiang Guo, Haozhe Huang, Yueli Xie, Chenlu Xu, Yuan Liu,* and Weihong Tan

Table S1. Characterization of Fe_3_O_4_@SiO_2_ NPs and Fe_3_O_4_@SiO_2_ NPCs (5% - 50%) by DLS.

| Sample | Hydrodynamic diameter (nm)^a^ | Z-potential (mV) ^a^ | Polydispersity index |
| --- | --- | --- | --- |
| Bare Fe_3_O_4_@SiO_2_ NPs | $\text{208±0.7}$ | $\text{-44.4±0.1}$ | 0.034 |
| Fe_3_O_4_@SiO_2_ NPC (5%) | $\text{298.3±0.9}$ | $\text{-30.6±0.2}$ | 0.145 |
| Fe_3_O_4_@SiO_2_ NPC (10%) | $\text{281.5±1.7}$ | $\text{-31.1±0.2}$ | 0.106 |
| Fe_3_O_4_@SiO_2_ NPC (25%) | $\text{283.0±3.8}$ | $\text{-29.7±0.4}$ | 0.059 |
| Fe_3_O_4_@SiO_2_ NPC (50%) | $\text{287.4±2.4}$ | $\text{-29.9±0.2}$ | 0.090 |

^a)^Data was shown as mean ± S.D., n = 3.


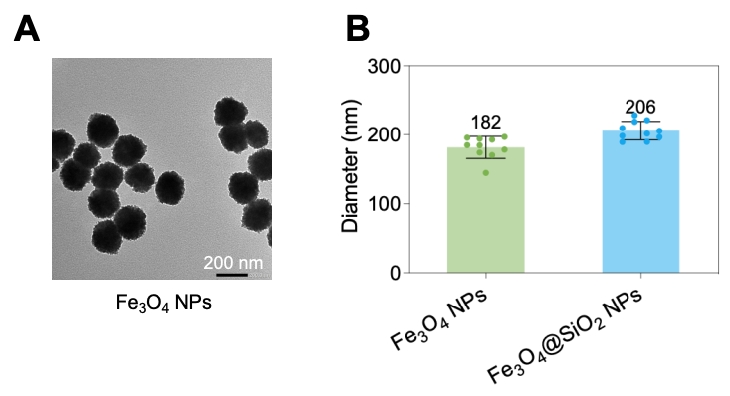


Figure S1. Characterization of NPs formation by TEM. (A) TEM image of Fe_3_O_4_ NPs. Scale bar = 200 nm. (B) Diameter of bare Fe_3_O_4_ NPs and Fe_3_O_4_ @SiO_2_ NPs. Mean ± S.D., n = 10.


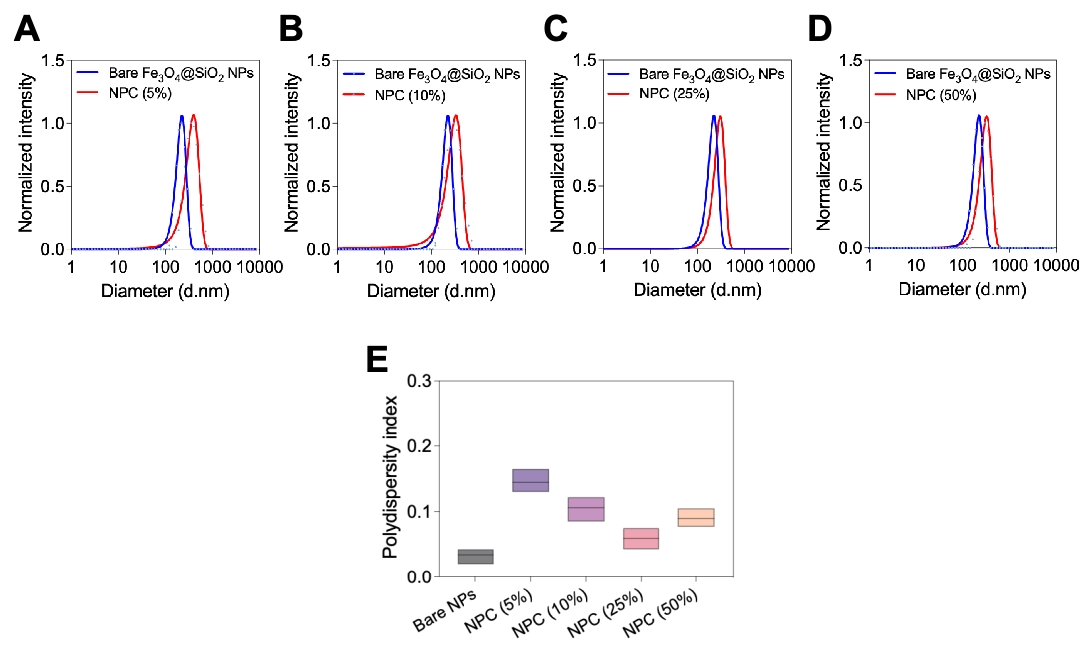


Figure S2. Hydrodynamic diameter of bare Fe_3_O_4_ @SiO_2_ NPs and Fe_3_O_4_ @SiO_2_ NPCs. Size measurement of Fe_3_O_4_ @SiO_2_ NPs before and after incubating with 5% (A), 10% (B), 25% (C), and 50% (D) serum. Intensity is normalized with the largest value in each dataset representing 1. (E) Polydispersity index of DLS measurement. Mean ± S.D., n = 3.


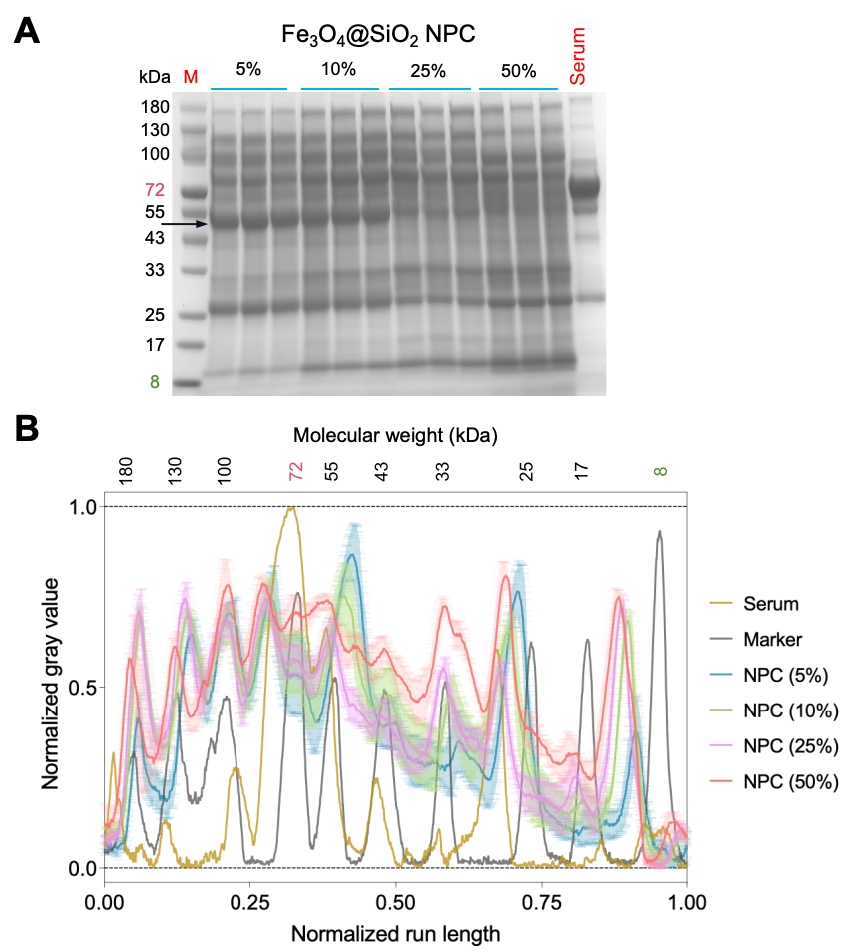


Figure S3. SDS-PAGE analysis of Fe_3_O_4_@SiO_2_ NPCs. (A) SDS-PAGE gel of proteins retrieved from Fe_3_O_4_@SiO_2_ NPCs of different serum concentrations. The first lane (M) was the molecular weight (MW) standard and the last lane was a control of pure serum. Three replicates were shown (n = 3). Serum concentration was indicated. (B) Semiquantitative densitometry analysis of SDS-PAGE gel in (A). Normalized gray values of Fe_3_O_4_@SiO_2_ NPCs are shown as Mean ± S.D., n = 3. Normalized run length and MW were labeled at the bottom and the top, respectively. Normalization: 0 and 1 are defined as the smallest and the largest value in the dataset, respectively.


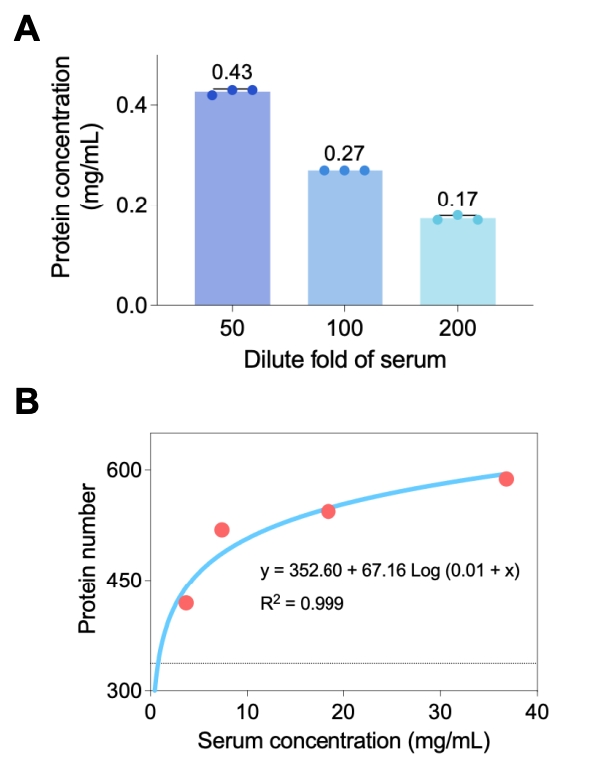


Figure S4. Serum concentration-dependent proteomic profiling. (A) Measurement of serum protein concentration using the BCA method. Pure serum was diluted 50, 100, and 200 times for accuracy. Mean ± S.D., n = 3. Averaged value from three dilute groups was used for subsequent fitting in (B). (B) Fitted curve of protein number identified in Fe_3_O_4_@SiO_2_ NPCs (5% - 50%) as a function of serum concentration based on the regression of Y ≈ a × Log (X + 0.01) + k.


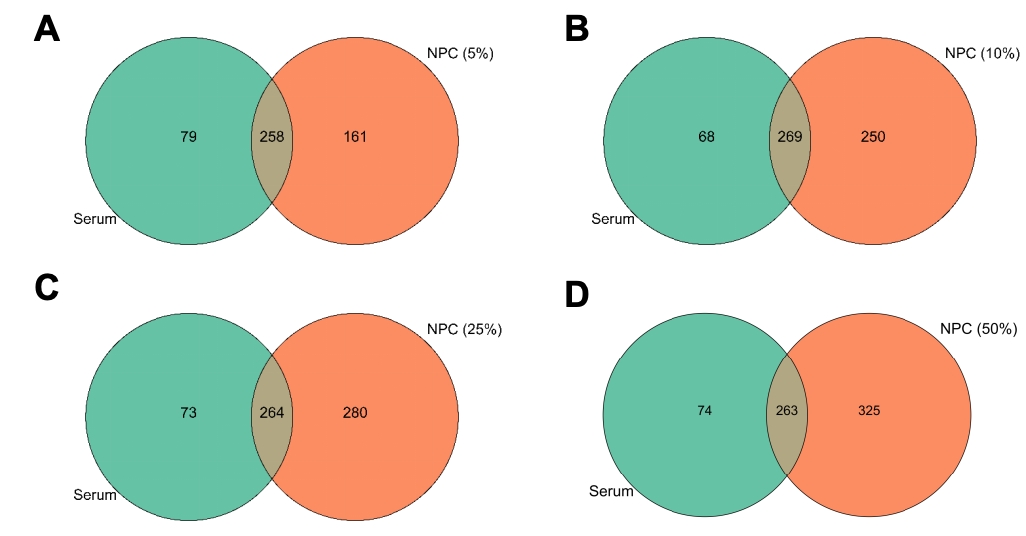


Figure S5. Similarity analysis of Fe_3_O_4_@SiO_2_ NPC profile and pure serum. Overlap of proteins identified in pure serum and Fe_3_O_4_@SiO_2_ NPC of 5% (A), 10% (B), 25% (C), and 50% (D) serum concentration, respectively.


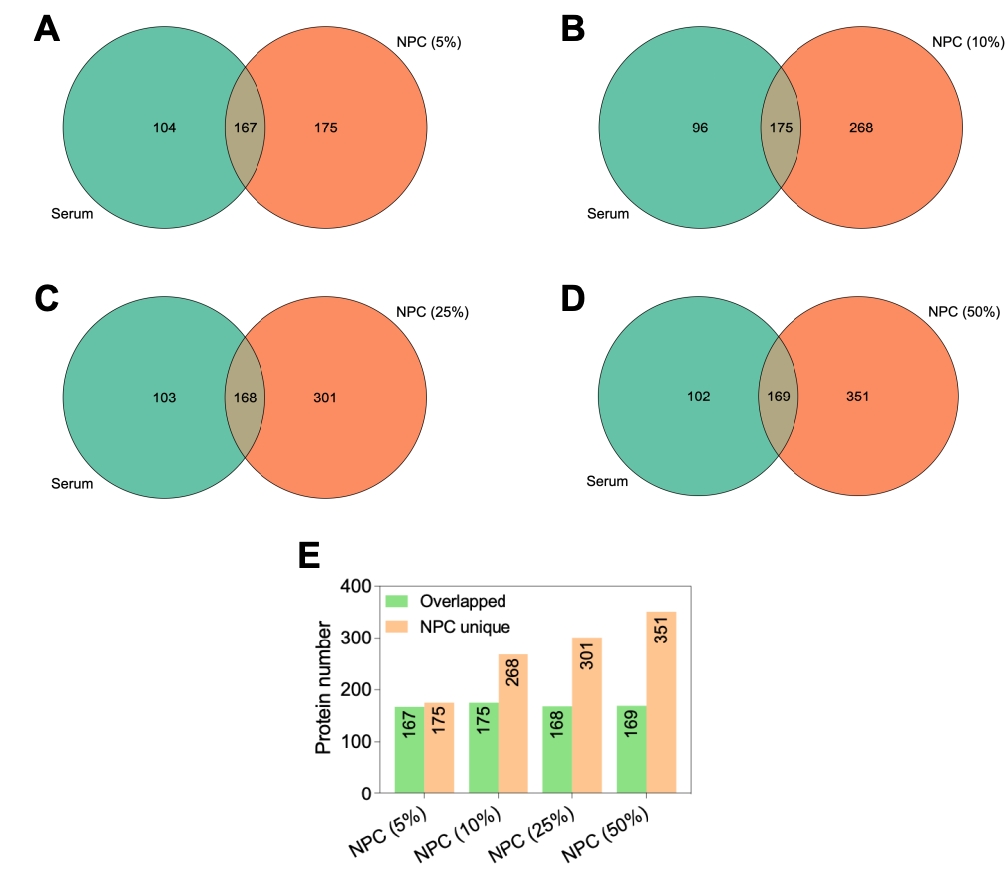


Figure S6. Serum concentration-dependent number of low-abundance proteins in Fe_3_O_4_@SiO_2_ NPC. (A-D) Number of overlapped low-abundance (< 0.1%) proteins between pure serum and Fe_3_O_4_@SiO_2_ NPC of 5% (A), 10% (B), 25% (C), and 50% (D) serum concentration, respectively. (E) Number of NPC unique proteins and overlapped proteins between pure serum and each NPC.


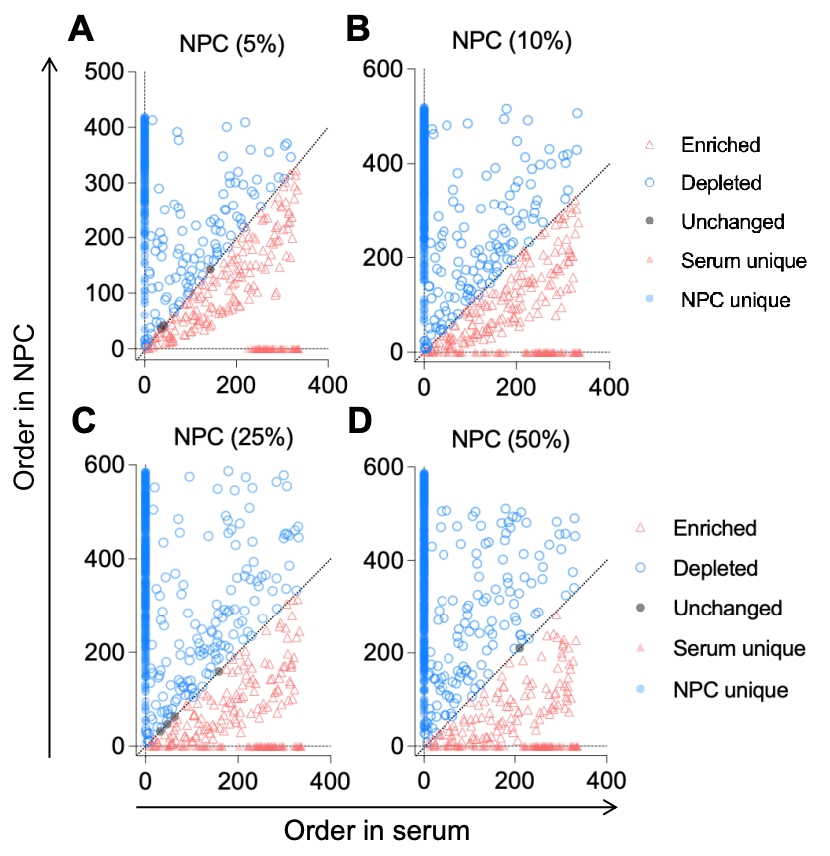


Figure S7. Investigation of serum protein depletion and enrichment in Fe_3_O_4_@SiO_2_ NPCs of different serum concentrations. Protein order in pure serum versus protein order in Fe_3_O_4_@SiO_2_ NPC of 5% (A), 10% (B), 25% (C), and 50% (D) serum concentration, respectively. Grey points on the diagonal (Y=X) indicated proteins with the same order in serum and in Fe_3_O_4_@SiO_2_ NPC. Red hollow triangles between the diagonal and X-axis indicated enriched proteins. Blue hollow circles between the diagonal and Y-axis indicated depleted proteins. Red triangle population on the X-axis represented unique proteins in pure serum. Blue points population on the Y-axis represented unique proteins in Fe_3_O_4_@SiO_2_ NPC.


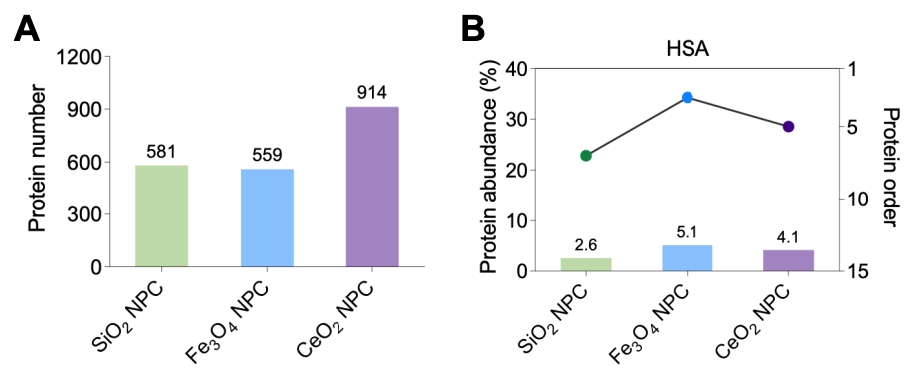


Figure S8. Investigation of the impact of material of nanomaterials on NPC profiling. (A) Number of proteins identified in NPCs of SiO_2_ NPs, Fe_3_O_4_ NPs, and CeO_2_ NPs. (B) Protein abundance (left axis; bar plot) and order (right axis; dot plot) of HSA in above NPCs, respectively.


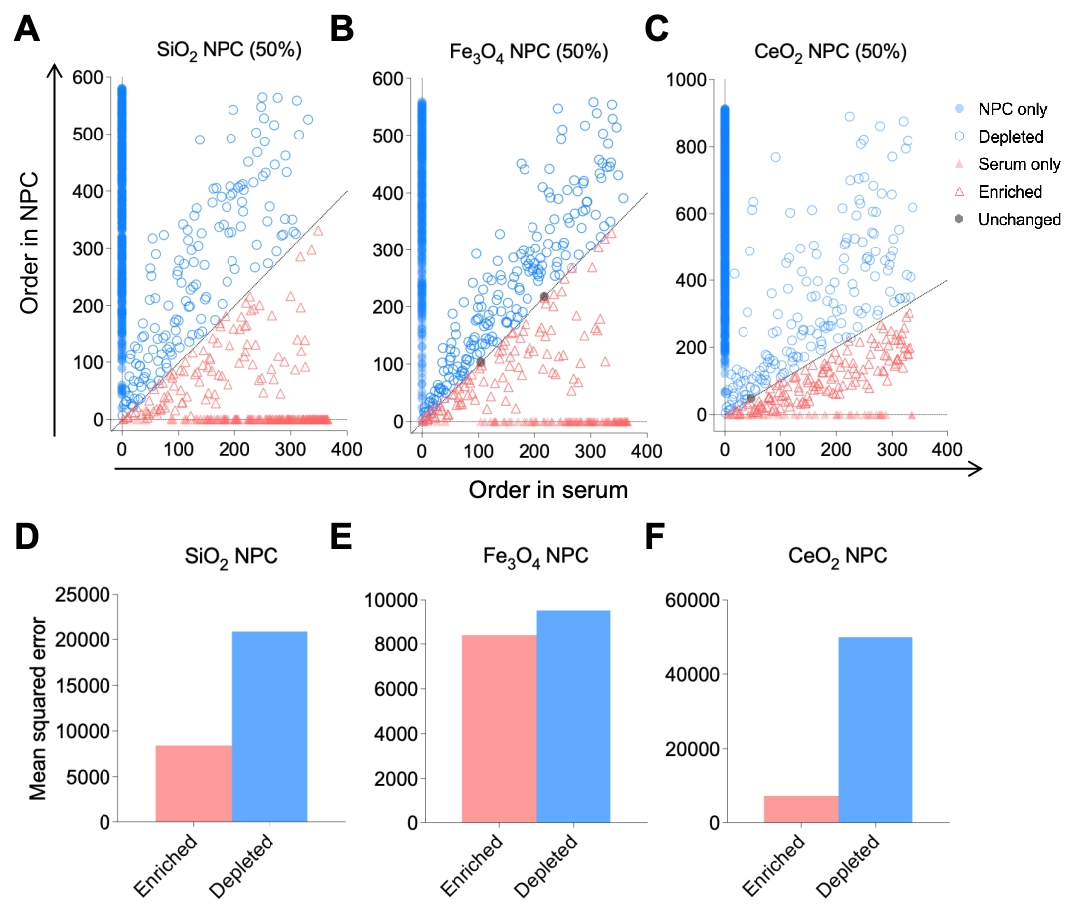


Figure S9. Measurement of serum protein enrichment and depletion ability of other three nanomaterials. (A-C) Protein order in pure serum versus protein order in NPC (50%) of SiO_2_ NPs (A), Fe_3_O_4_ NPs (B), and CeO_2_ NPs (C). Grey points on the diagonal (Y = X) indicated proteins stay the same order in both serum and each NPC. Red hollow triangles between the diagonal and X-axis indicated enriched proteins while blue hollow circles between the diagonal and Y-axis indicated depleted proteins. Red triangles population on the X-axis represented proteins identified only in serum. Blue points population on the Y-axis represented proteins identified only in each NPC. (D-F) Quantitative characterization of protein enrichment or depletion ability of above NPCs of SiO_2_ NPs (D), Fe_3_O_4_ NPs (E), and CeO_2_ NPs (F).


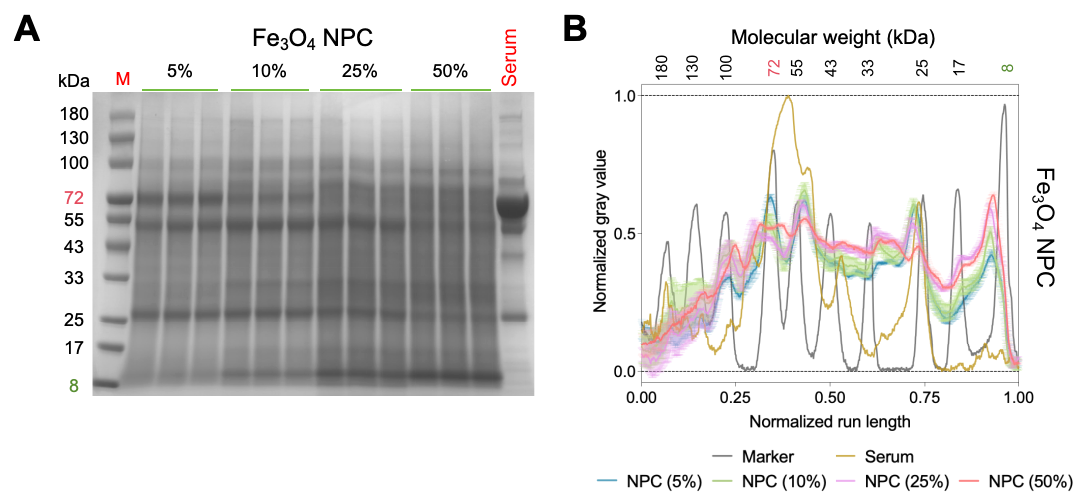


Figure S10. SDS-PAGE analysis of Fe_3_O_4_ NPCs (5% - 50%). (A) SDS-PAGE gel of proteins retrieved from Fe_3_O_4_ NPCs of different serum concentrations. The first lane (M) was the MW standard and the last lane was a control of pure serum. Three replicates were shown (n = 3). Serum concentration was indicated. (B) Semiquantitative densitometry analysis of SDS-PAGE gel in (A). Normalized gray values of NPCs are shown as Mean ± S.D., n = 3. Normalized run length and MW were labeled at the bottom and the top, respectively. Normalization: 0 and 1 are defined as the smallest and the largest value in the dataset, respectively.


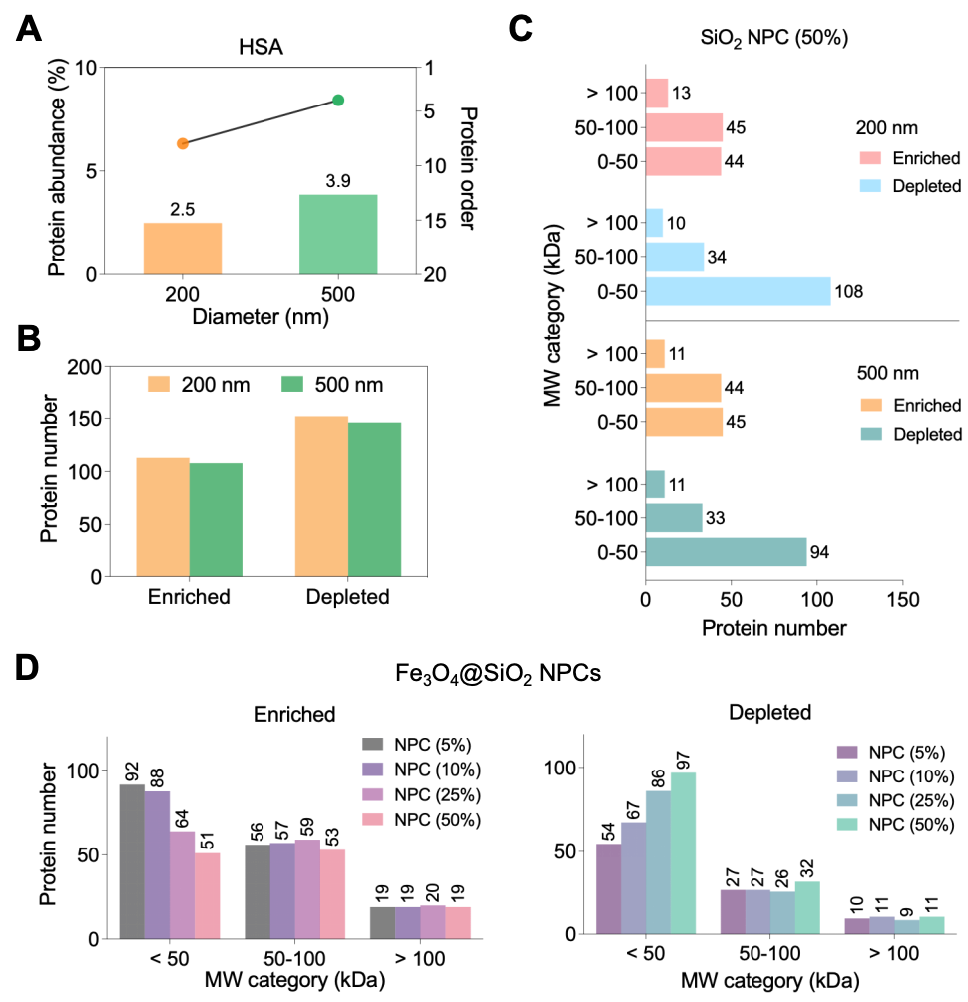


Figure S11. The effect of NP size on NPC formation and profiling. (A) Protein abundance (left axis; bar plot) and order (right axis; dot plot) of HSA in NPCs of SiO_2_ NPs with 200 nm and 500 nm diameters. (B) Distribution of enriched and depleted proteins in NPCs of SiO_2_ NPs with different diameters. (C) MW Distribution of enriched and depleted proteins in above SiO_2_ NPCs. (D) MW Distribution of enriched and depleted proteins in Fe_3_O_4_@ SiO_2_ NPCs of different serum concentrations.


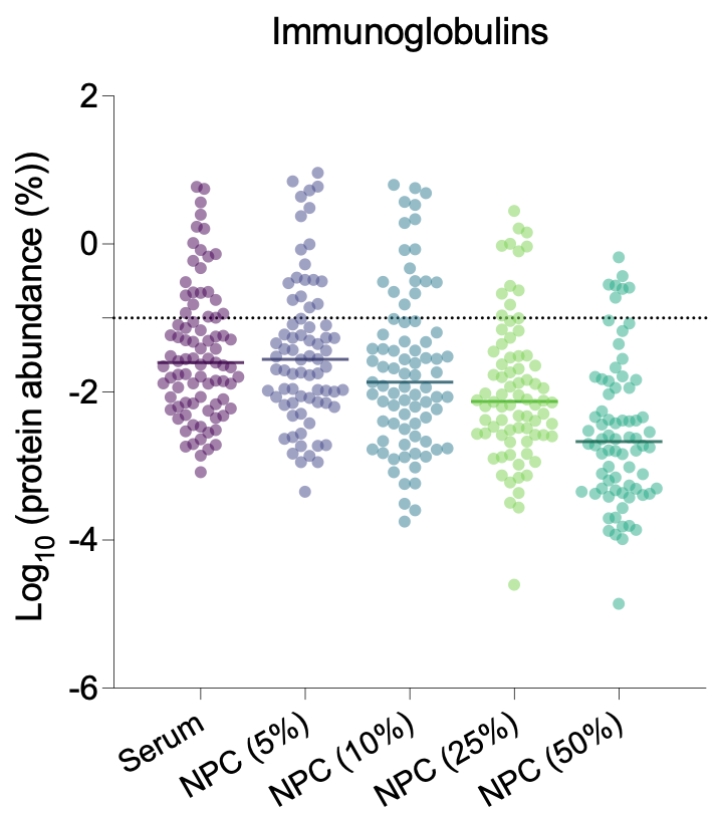


Figure S12. Distribution of immunoglobulin abundance in pure serum and Fe_3_O_4_@SiO_2_ NPCs of different serum concentrations. Dashed line indicated Y = 0.1%. Line represents median.


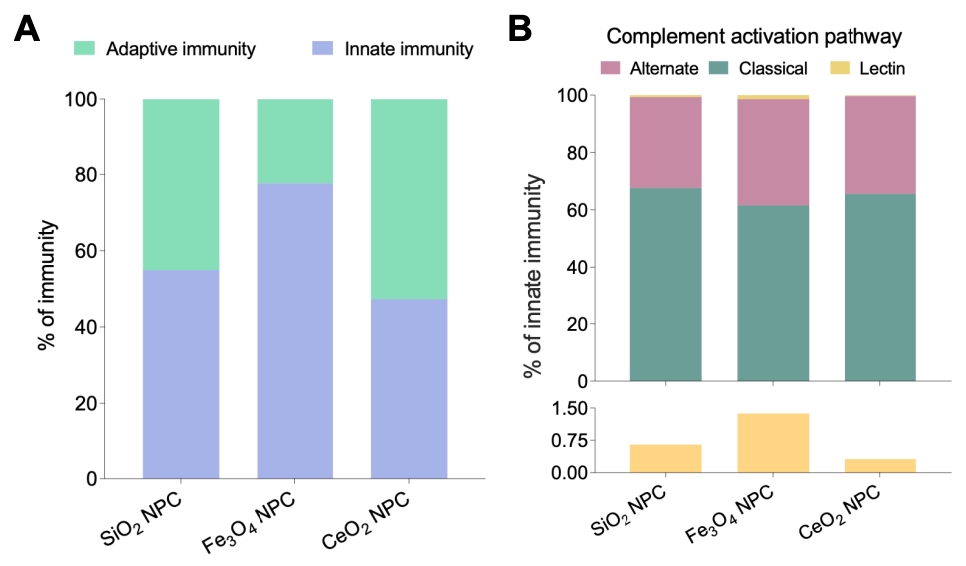


Figure S13. Profiling of immune-related proteins of NPCs (50%) of different nanomaterials. (A) Composition analysis of adaptive and innate immunity in NPCs of SiO_2_ NPs, Fe_3_O_4_ NPs, and CeO_2_ NPs. (B) Composition analysis of innate immunity (complement activation pathway) in NPCs of SiO_2_ NPs, Fe_3_O_4_ NPs, and CeO_2_ NPs.


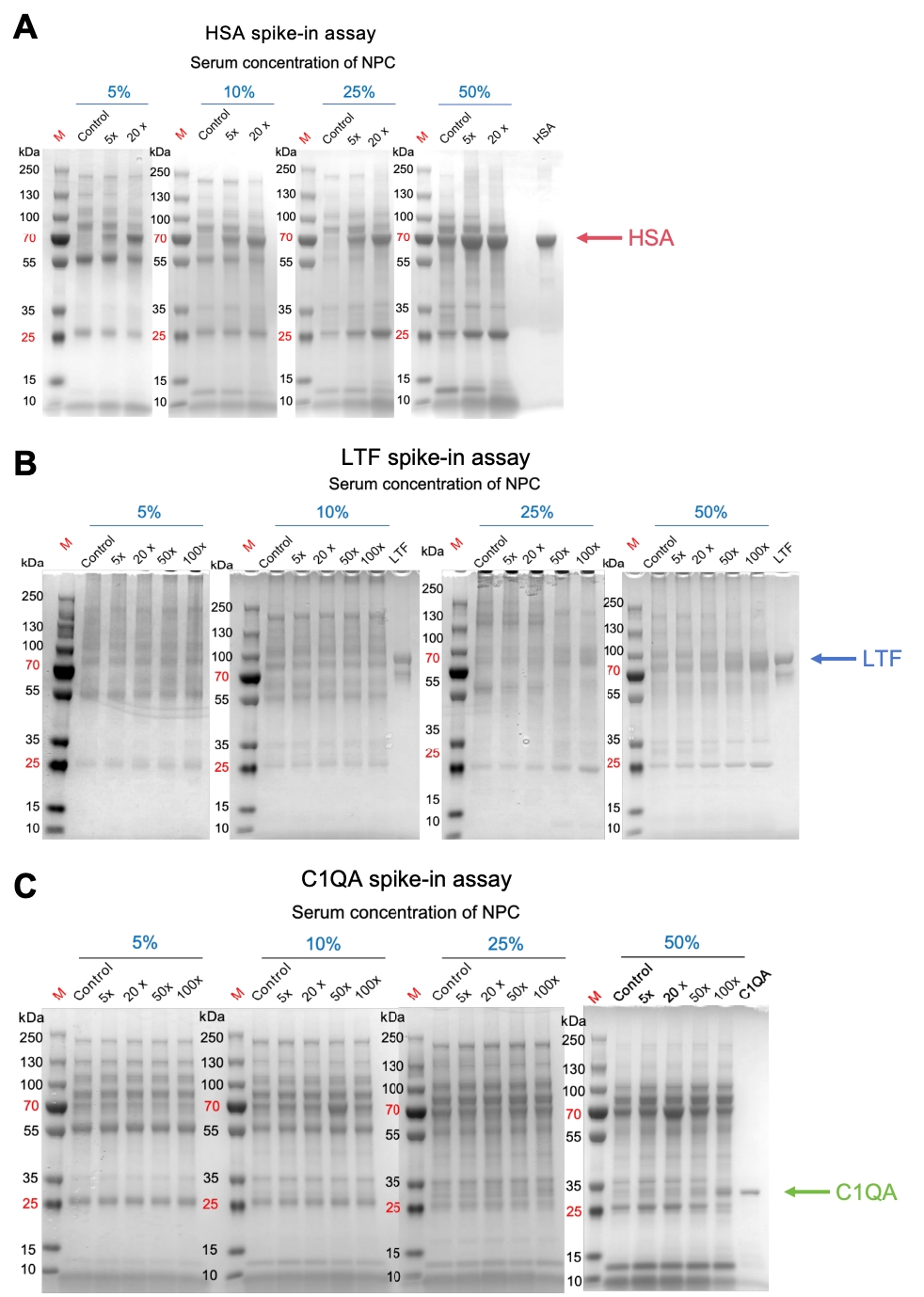


Figure S14. Characterization of Fe_3_O_4_@SiO_2_ NPCs from spike-in assays. SDS-PAGE gel of proteins retrieved from Fe_3_O_4_@SiO_2_ NPCs (5% - 50%) of spike-in assays of HSA (A), LTF (B), and C1QA (C). Spike-in folds of HSA were 5 and 20, while spike-in folds of LTF and C1QA were 5, 20, 50, and 100. Fe_3_O_4_@SiO_2_ NPC without spike-in protein was control. The first lane (M) was the MW standard and the last lane was a control of HSA (A), LTF (B), and C1QA (C).


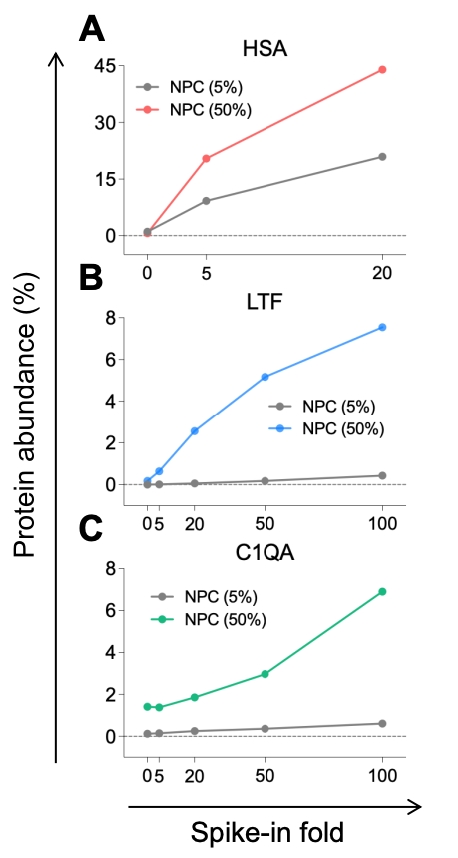


Figure S15. Protein concentration attributes to its abundance in Fe_3_O_4_@SiO_2_ NPC. Protein abundance (%) of HSA (A), LTF (B), and C1QA (C) in NPCs of corresponding spike-in assays. Serum concentration was indicated. Dashed line indicated Y=0.


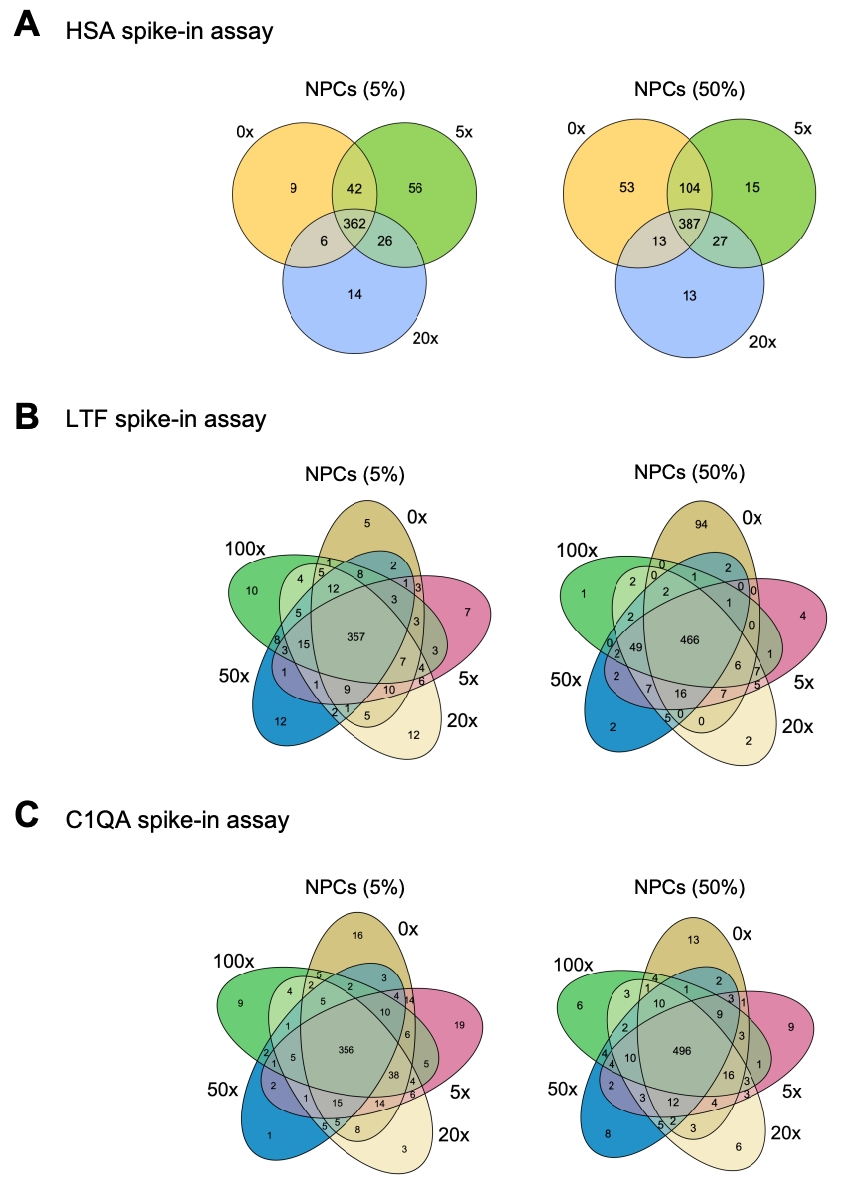


Figure S16. Similarity analysis of Fe_3_O_4_@SiO_2_ NPC profile from spike-in assay. Venn diagram of proteins identified in NPCs of HSA (A), LTF (B), and C1QA (C) spike-in assay. Spike-in fold and serum concentration were indicated.


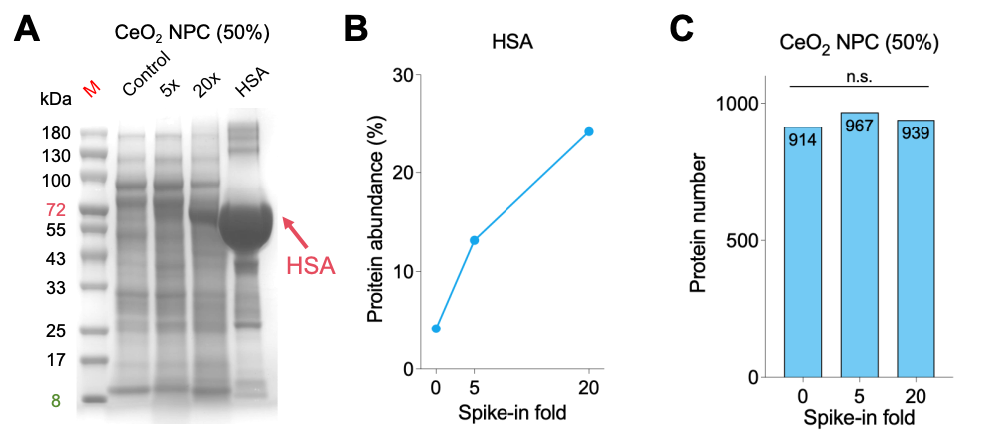


Figure S17. Intrinsic tolerance of CeO_2_ NPCs (50%) to ultra-high abundant protein. (A) SDS-PAGE gel of proteins retrieved from CeO_2_ NPCs (50%) of spike-in assays of HSA. Spike-in folds of HSA were 5 and 20. NPC without spike-in protein was control. The first lane (M) was the MW standard and the last lane was a control of HSA. (B) Change of protein abundance of HSA in NPCs with or without spiking-in HSA. (C) Number of proteins identified in CeO_2_ NPCs (50%) of HSA spike-in assay. P-values are generated from Wilcoxon sighed-rank test. n.s. denotes non significance (P > 0.05).


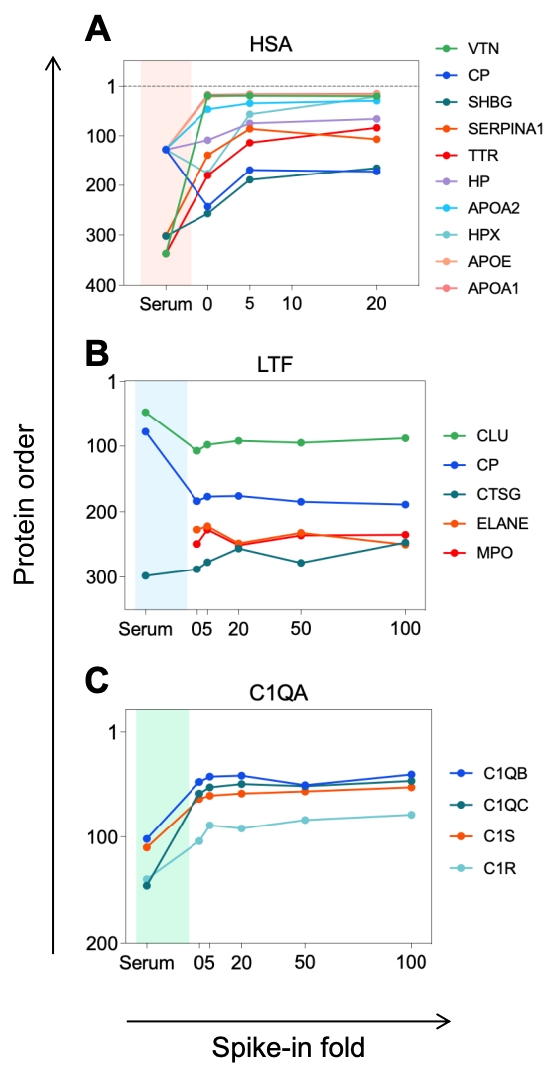


Figure S18. Investigation of coronal protein-protein interactions (PPI) mediated by spike-in protein in Fe_3_O_4_@SiO_2_ NPCs (50%). Order change of proteins associated with HSA (A), LTF (B), and C1QA (C) in pure serum and NPCs (5%) before and after spiking corresponding protein.


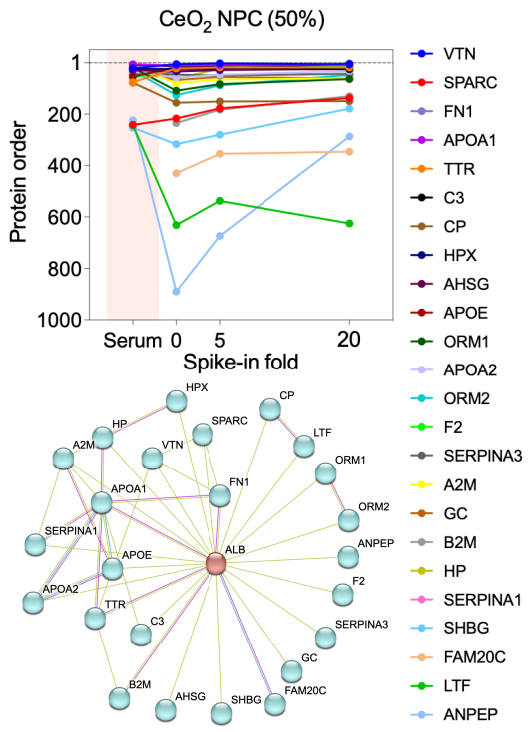


Figure S19. HSA-mediated PPI in corona proteins of CeO_2_ NPCs of HSA spike-in assay. Order change of proteins associated with HSA (upper) and related physical PPI subnetwork (lower). PPI was generated using the STRING database. Minimum required interaction score = 0.700.


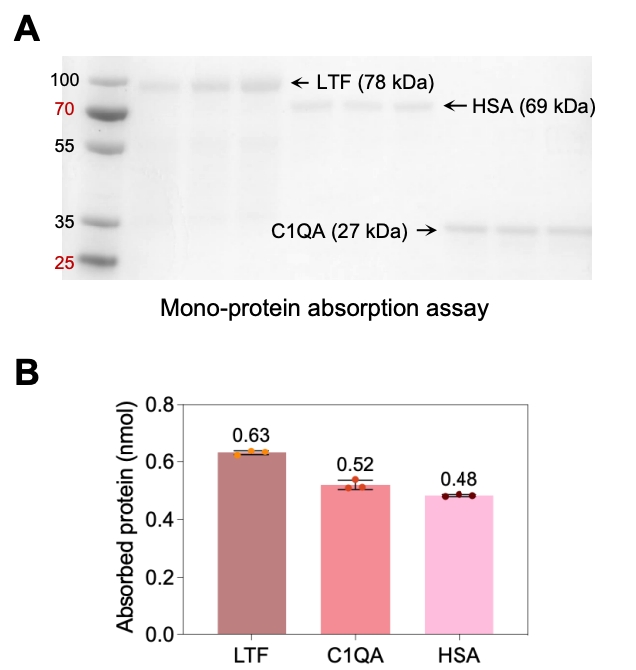


Figure S20. Mono-protein assay. (A) SDS-PAGE gel of proteins retrieved from Fe_3_O_4_@SiO_2_ NPCs of mono-protein assays. The first lane was the MW standard. Arrows indicate the input protein and its MW. Three replicates were shown. (B) Amounts of absorbed protein in Fe_3_O_4_@SiO_2_ NPCs. Data represents as Mean ± S.D., n = 3.
